# Long-term exercise enhances meningeal lymphatic vessel plasticity and drainage in a mouse model of Alzheimer's disease

**DOI:** 10.1186/s40035-025-00497-2

**Published:** 2025-07-25

**Authors:** Yan Chen, Jiachen Cai, Yuzhu She, Xiaoxin He, Hu Feng, Xuewei Li, Yiran Wei, Yi Fan, Wen-e Zhao, Mengmei Yin, Linjuan Yuan, Yuxi Jin, Fengfei Ding, Chengyu Sheng, Junying Gao, Qian Li, Ming Xiao

**Affiliations:** 1 Jiangsu Key Laboratory of Neurodegeneration, Nanjing Medical University, Nanjing, 211166 China; 2 Institute of Brain Science and Brain-Inspired Research, Shandong First Medical University and Shandong Academy of Medical Sciences, Jinan, 250117 China; 3 Department of Neurology, Brain Institute, The Affiliated Nanjing Brain Hospital of Nanjing Medical University, Nanjing, 210029 China; 4 Department of Analytical and Testing Center, Nanjing Medical University, Nanjing, 211166 China; 5 Department of Neurology, The First Affiliated Hospital of Nanjing Medical University, Nanjing, 210029 China; 6 Department of Pharmacology, School of Basic Medical Sciences, Fudan University, Shanghai, 200032 China; 7 Department of Anatomy, Nanjing Medical University, Nanjing, 211166 China

**Keywords:** Alzheimer's disease, Lymphangiogenesis, Meningeal lymphatics, Treadmill exercise, EAF2-p53-TSP-1

## Abstract

**Background:**

Meningeal lymphatic drainage is crucial for the clearance of amyloid β (Aβ), supporting the maintenance of brain homeostasis. This makes it a promising therapeutic target for Alzheimer's disease (AD). Long-term exercise can reduce the risk of AD; however, the underlying mechanism is not fully understood. In this study, we investigated whether exercise alleviates AD-related pathological changes by improving meningeal lymphatic drainage and its potential mechanisms.

**Methods:**

The morphological and functional features of meningeal lymphatic vessels, as well as Aβ and reactive gliosis in the brain, were compared between 6.5-month-old 5 × FAD mice with or without 1 month of treadmill exercise. RNA sequencing, protein interactions analysis, gene knockdown mediated by adeno-associated virus, and lymphatic endothelial cell culture were conducted to investigate the mechanism underlying exercise-induced meningeal lymphatic vessel plasticity in 5 × FAD mice.

**Results:**

The structural integrity of meningeal lymphatic vessels was compromised in 5 × FAD mice, compared with the wild-type mice. Treadmill exercise increased the diameter and the drainage capacity of the meningeal lymphatic vessels, reduced Aβ deposition, reactive gliosis and astrocyte senescence in the hippocampus and frontal cortex, and improved cognitive function of 5 × FAD mice. Mechanistically, thrombospondin-1 (TSP-1) exacerbated the inhibitory effect of Aβ on lymphatic vessel formation and plasticity through interactions with CD36 and CD47, respectively. Exercise decreased the expression of TSP-1 in reactive astrocytes of AD mice by downregulating eleven-nineteen lysine-rich leukemia-associated factor 2 (EAF2), a protein that facilitates the transcription of the TSP-1-encoding gene *Thbs-1* by binding p53. Ultimately, we found that hippocampal astrocyte-specific knockdown of *Thbs-1* or *Eaf2* enhanced meningeal lymphatic drainage and alleviated AD-like pathology in the hippocampus of 5 × FAD mice.

**Conclusions:**

Long-term exercise protects against AD by enhancing the plasticity and drainage of meningeal lymphatic vessels through downregulation of the EAF2–p53–TSP-1 pathway associated with reactive astrocytes.

**Supplementary Information:**

The online version contains supplementary material available at 10.1186/s40035-025-00497-2.

## Introduction

Alzheimer's disease (AD) is one of the most common age-dependent neurodegenerative diseases, posing a serious threat to the health and life of older adults. Although monoclonal antibodies such as aducanumab and lecanemab can specifically reduce the deposition of amyloid β (Aβ) plaques, their long-term efficacy and potential complications still present challenges [1]. It is known that the imbalance between the production and clearance of Aβ occurs prior to the onset of cognitive impairment, causing excessive aggregation of Aβ and a series of neuropathological cascades [2]. Notably, a large number of astrocytes are persistently activated and undergo senescence, losing their ability to maintain brain homeostasis and exhibiting the senescence-associated secretory phenotype, which in turn accelerates neurodegeneration [3, 4]. Therefore, timely and effective clearance of Aβ from the brain and prevention of astrocyte senescence may be beneficial for delaying or even preventing the onset of AD [5, 6].

Meningeal lymphatic vessels have recently been characterized in humans and rodents [7]. They mediate the drainage of macromolecular waste [8, 9], cellular debris [10], neurotropic viruses [11], and brain tumor cells [12] from the brain. Meningeal lymphatic drainage progressively deteriorates during natural aging and in AD [13–15]. Blocking meningeal lymphatic vessels exacerbates Aβ load and memory deficits in transgenic mouse models of AD [8, 14]. Therefore, enhancing meningeal lymphatic drainage could be a novel therapeutic target for AD.

Notably, the specialized morphological features of meningeal lymphatic vessels are associated with their drainage functions [15]. A continuous zipper-like vascular endothelial (VE)–cadherin junction and a discontinuous button-like junctional pattern in lymphatic endothelial cells (LECs) are associated with distinct modes of cerebrospinal fluid (CSF) macromolecule transport facilitated by meningeal lymphatic vessels. Zipper-like junctions form tight, continuous barriers that support directional fluid flow, while button-like junctions are more permissive and facilitate macromolecule uptake. Balance between these junction types is crucial for efficient CSF drainage and is disrupted in aged mice, potentially contributing to impaired brain waste clearance [15]. Overexpression of vascular endothelial growth factor C (VEGFC) induces meningeal lymphangiogenesis [16], whereas inhibitors of lymphangiogenesis, such as pigment epithelium-derived factor, suppress peripheral nasopharyngeal lymphangiogenesis [17]. Nonetheless, the mechanisms that underlie the impairment of the integrity and plasticity of meningeal lymphatic vessels during the progression of AD remain unclear.

Exercise intervention is one of the most effective nonpharmacologic therapeutic modalities, exerting beneficial effects on various organs, particularly the brain [18, 19]. For example, long-term exercise not only enhances the production of brain-derived neurotrophic factor [20] and synaptic plasticity [21], but also diminishes oxidative stress [22] and age-related gliosis [23] in the brain. Additionally, recent evidence suggests that the astrocytic aquaporin 4 (AQP4)-mediated glymphatic transport plays a role in the neuroprotective effects of voluntary exercise [24–26]. Nonetheless, it remains undetermined whether exercise can enhance meningeal lymphatic plasticity during the progression of AD.

Here, we investigated the role of astrocyte-derived factors in the regulation of meningeal lymphatic vessel function in a 5 × FAD transgenic mouse model of AD. We further examined how long-term physical exercise influences astrocyte activity, meningeal lymphatic drainage, and Aβ clearance. By integrating genetic and behavioral interventions, our work aimed to identify novel astrocyte-derived modulators of lymphangiogenesis and to explore the therapeutic potential of exercise in ameliorating Aβ-associated neuropathology and cognitive impairment.

## Methods

### Animals

B6.Cg-Tg (APPSwFlLon, PSEN1*M146L*L286V) 6799Vas/Mmjax (5 × FAD, strain# 008730) mice were obtained from Jackson Laboratories (Bar Harbor, ME). The mice, along with their age-matched wild-type (WT) littermates, were housed in controlled ambient temperatures and exposed to a 12-h light/12-h dark cycle, with free access to standard rodent chow and clean water. Each group of mice consisted of equal numbers of males and females. All animal experiments were approved by the Animal Care and Use Committee of Nanjing Medical University (IACUC-1812054).

### Treadmill exercise training

5 × FAD mice aged 5.5 months were randomly assigned to either a treadmill exercise group or a sedentary group. The treadmill exercise was applied twice a day (at 09:00 and 20:00, respectively), for one month. Mice were acclimated and trained on a 10° uphill treadmill, beginning with 30 min of running at 8 m/min, followed by 30 min at 10 m/min for the first two days as a warm-up. Starting on the third day, mice were subjected to increasing treadmill speeds, with increments of 1 m/min every 20 min, for a total of 90 min per session [27]. Mice in the sedentary control group were kept in identical conditions but remained in their natural state. After one month of repeated training, the animals underwent behavioral tests followed by pathological analyses.

### In vivo two-photon imaging

After 10 days of treadmill exercise training, mice were anesthetized with an intraperitoneal injection of a mixed ketamine (80 mg/kg) and xylazine (8 mg/kg) in saline, and secured in a stereotaxic device. The hair was shaved from head to neck and skin was cleaned with iodine and 75% ethanol. Following a surgical skin incision above the parietal region, the skull bone was thinned using an electrical micro drill. A total of 3 μL of A488-Lyve-1 (Invitrogen, Carlsbad, CA; Cat# 53–0443-82) was injected into the cisterna magna within 5 min via a 5 μL Hamilton syringe. The syringe was left in place for an additional 5 min and then withdrawn slowly. After suturing the exposed incision, the mice were returned to their home cage. Twenty-four hours later, they received an intrahippocampal injection (anteroposterior − 2.0 mm, mediolateral ± 1.8 mm, dorsoventral − 2.0 mm) of 1 μL of Fluor 555-labeled Aβ (1 mg/mL, AnaSpec, Fremont, CA; Cat# AS-60480–01). One hour later, in vivo two-photon imaging was performed. Mouse head was fixed on a metal holder to minimize movement during live imaging. As previously described [11], a confocal scanning system (Zeiss ZEN) equipped with a two-photon laser scanning microscope and a 20 × water-immersion lens installed on an upright microscope (Zeiss LSM880, Germany) was used for imaging.

### AAV-mediated knockdown of *Thbs-1* or *Eaf2* in astrocytes

rAAV-GFaABC1D-mCherry-5′miR-30a-shRNA (Thbs1)-3′miR-30a-WPREs and rAAV-GFaABC1D-EGFP-5′miR-30a-shRNA (Eaf2)-3′miR-30a-WPREs were obtained from BrainVTA (Wuhan, China). The shRNA sequences verified to efficiently knockdown mouse *Thbs-1* or *Eaf2* were used: siRNA *Thbs1*, 5′-GAUGACUACGCUGGCUUUGUU-3′, siRNA *Eaf2*, 5′-GGACUUCCAAUCUUGUACATT-3′. As described above, after anesthetization, 5-month-old WT and 5 × FAD mice were secured in a stereotaxic apparatus. rAAV2/5-GFaABC1D-Thbs1-shRNA-mCherry, rAAV2/9-GFaABC1D-scramble-shRNA-mCherry, rAAV2/5-GFaABC1D-Eaf2-shRNA-EGFP, or rAAV2/5-GFaABC1D-scramble-shRNA-EGFP was injected into the bilateral hippocampal region (anteroposterior − 2.0 mm, mediolateral ± 1.8 mm, dorsoventral − 2.0 mm, 1 µL for each hemisphere). Four weeks after virus injection, mouse behavior and pathology were evaluated.

### Y-maze test

The Y maze (27 cm × 9 cm × 24 cm) consisted of three arms. During the training stage, each mouse was placed in the start arm to explore for 5 min, with the novel arm being blocked. Two hours later, the mouse was allowed to freely explore the entire maze for 5 min. The percentage of time spent in the novel arm and the number of entries into the novel arm were recorded by the video tracking software (TopScan, CleverSys, Inc., Reston, VA).

### Novel object recognition (NOR) test

The NOR test was conducted in two phases: familiarization and testing [10]. During the familiarization phase, each animal was permitted to freely explore an open arena (40 cm × 40 cm × 30 cm) for 5 min, in which two identical objects were placed in opposite diagonal corners. In the testing phase 2 h later, one familiar object was replaced by a novel object (differing in color and shape), and the mice were allowed to explore the arena for another 5 min. Exploration of objects was defined as mouse sniffing or interacting with an object from within 2 cm and was quantified using a video tracking software (TopScan, CleverSys, Inc.). The discrimination index was calculated as follows: discrimination index = (*T*_novel_-*T*_familiar_)/(*T*_novel_ + *T*_familiar_).

### Elevated plus maze (EPM) test

The EPM consisted of two open arms (35 cm × 6 cm) and two closed arms (35 cm × 6 cm × 15 cm), each elevated 75 cm above the floor. Mice were placed in the central hub and allowed to freely explore the maze for 5 min. A video tracking software (TopScan, CleverSys, Inc.) was utilized to quantify the time spent in the open arms and the frequency of entries into the open arms.

### Primary astrocytes and cell line cultures and treatment

Primary astrocytes were isolated from neonatal mice as previously described [10]. In brief, after removing the meningeal vessels, the hippocampus and surrounding cortices were microdissected and subjected to trypsin digestion. Tissue homogenates were passed through a 70-μm mesh filter, resuspended in astrocyte growth medium (DMEM (Gibco, Carlsbad, CA; Cat# 11960) containing 10% FBS and 100 U/mL penicillin/streptomycin (Gibco, Cat# 15–140-122), and plated on 10-cm petri dishes. The cells were incubated at 37 °C in 5% CO_2_. The medium was fully replaced every 3 to 4 days, and cells were passaged using 0.05% Trypsin (Gibco, Cat# 25–200-056) when they reached full growth.

For experiments involving treatment of primary astrocytes with Aβ_1-42_ oligomers, primary astrocytes (12–15 days in vitro, DIV 12–15) were transferred to complete serum medium and then incubated in the presence or absence of oligomeric Aβ_1-42_ (at concentrations of 0, 5, and 10 μmol/L) for 48 h. Some experiments continued with transfection of siR-Eaf2, after which the pellet and supernatant were collected 72 h later.

Cultures of the *Mus musculus* lymphoid endothelial cell line (SVEC4-10) (ATCC, Cat# CRL-2181), human aortic endothelial cells (HAECs) and human-derived lymphatic endothelial cell line (HLEC) were routinely maintained in DMEM medium supplemented with 10% FBS and 100 U/mL penicillin/streptomycin. The SVEC4-10 cells were planted on a 24-well plate and then treated with a gradient concentration of recombinant TSP-1 protein (R&D Systems, Minneapolis, MN; Cat# 3074-TH-050) or oligomer Aβ_1-42_. To investigate the effect of the TSP-1–CD47 pathway on junctional patterns in vitro, SVEC4-10 cells were pretreated with siR-CD47 before treatment with recombinant TSP-1. The sequences were as follows:

siR-Eaf2, 5′-CAAAGGCUGCUCCAGCUCUdTT-3′;

siR-Cd47 (Mus), 5′- CUUGCAUCGUCCGUAAUGUTT-3′;

Negative control (NC), 5′-UUCUCCGAACGUGUCACGUTT-3′.

### CSF and tissue collection

After anesthesia, CSF was collected from the cisterna magna using a borosilicate glass pipette with an internal filament, and centrifuged at 1000 × *g* for 15 min. The supernatant was carefully transferred to a collection tube and stored at − 80 °C. Subsequently, the deep cervical lymph nodes (dCLNs) were removed and preserved in 4% paraformaldehyde (PFA). The mice were then transcardially perfused with ice-cold phosphate-buffered saline (PBS). Following the removal of the skin and muscle from the head, the hippocampi were collected and stored at − 80 °C until further processing. The brains and skullcaps were kept in 4% PFA for an additional 24 h. The fixed meninges, including the dura mater and arachnoid, were carefully dissected from the skullcaps using a stereomicroscope. Fixed brains and dCLNs were subsequently subjected to gradient dehydration using 20% and 30% sucrose solutions and embedded in tissue-plus OCT compound (Sakura, Torrance, CA; Cat# 4583). The brain segments encompassing the hippocampus and adjacent cerebral cortex were sliced into coronal sections at a thickness of 20 μm, while the frozen lymph nodes were sectioned into 10-μm slices using a cryostat (Leica, CM1950, Germany). The sections were then transferred to cryoprotectant (50% glycerol, 50% 0.1 mol/L PBS, pH 7.4) and stored at − 20 °C until needed. SVEC4-10 cells were fixed in 4% PFA for 15 min at room temperature, washed with PBS, and subsequently stored at 4 °C until further staining.

### Immunofluorescence

Frozen brain sections, lymph node slices, meningeal whole mounts, SVEC4-10 cells, and primary astrocytes on glass coverslips were blocked and permeabilized for one hour at room temperature using a block/stain buffer (0.3% Triton X-100 and 10% bovine serum in PBS). The sections were then incubated with primary antibodies (Table S1) in block/stain buffer overnight at 4 °C, washed three times in PBS, and incubated with secondary antibodies (at a 1:1000 dilution) for 2 h at room temperature. After being washed in PBS and incubated with 4',6-diamidino-2-phenylindole dihydrochloride (DAPI) at a concentration of 1 μg/mL in PBS, the sections were coverslipped until images were acquired using a wide-field microscope (DM4000B, Leica) or a confocal microscope (Zeiss LSM710, Germany).

### Thioflavin-S staining

Frozen brain sections were stained with 1% Thioflavin-S (Sigma, St. Louis, MO; Cat# 1326–12-1) for 5 min. After rinsing with distilled water, they were differentiated with 70% alcohol for 1 min. The sections were then washed with PBS and mounted with glass coverslips.

### Western blotting

Total proteins were extracted from primary astrocytes and mouse hippocampus by incubation in lysis buffer (25 mmol/L Tris pH 7.4, 150 mmol/L NaCl, 1 mmol/L CaCl_2_, 1 mmol/L MgCl_2_, 0.5% NP-40, and protease inhibitors) for 10 min on ice. The lysates were then centrifuged at 13,000 × *g* at 4 °C for 20 min, and the supernatant was collected. Protein concentration was determined using the BCA protein assay (Beyotime Biotechnology, Xiamen, China). Thirty micrograms of protein were mixed with 6 × loading buffer (Thermo Scientific, Waltham, MA) and boiled at 95 °C for 5 min. The samples were loaded onto 8%–15% gradient gel and transferred to PVDF membranes. The membranes were blocked with 5% non-fat milk for 1 h, and subsequently incubated with primary antibodies (Table S1) overnight at 4 °C. After washing the membranes three times with TBST, they were incubated with secondary antibodies for 1 h at room temperature. Finally, all bands were washed three times with TBST and imaged using an imaging system (ImageQuant™ LAS 4000 mini, version 1.2).

### ELISA

TSP-1 and EAF2 levels in the CSF and hippocampus of mice, as well as in the pellet and supernatant of primary astrocytes, were determined with ELISA commercial kits (Jingmei Co., Ltd. Cat# JM-04106H1, JM-13432M2) following the manufacturer’s instructions.

### RNA extraction and qRT-PCR

Total RNA was extracted using Trizol reagent (TaKaRa, Kusatsu, Japan), and cDNA was generated using a reverse transcriptase kit (Nanjing Vazyme Biotech Co., Ltd., Cat# R323) following the manufacturer’s instructions. qRT-PCR was performed on an ABI 7300 Fast Real-Time PCR System (Applied Biosystems, Foster City, CA) with qRT-PCR SYBR master mix (Vazyme Biotech Co., Ltd. Cat# Q712). mRNA expression level was calculated with the 2^−ΔΔCt^ method as described in a previous report [10]. GAPDH was used as an internal control. The primers used are listed in Table S2.

### Tube formation assay

Tube formation assay was performed to assess the effects of recombinant TSP-1 on the tube formation by murine and human LECs in vitro. Matrigel (Coring, NY, USA; Cat# 354243) (10 μL) was added to the inner wells of 15-well Ibidi μ-slides (Ibidi, Martinsried, Germany; Cat# 81506) and allowed to polymerize for one hour at 37 °C to form a gel-like surface. SVEC4-10 or HLECs in complete culture medium (DMEM, Gibco) with 10% FBS, 100 U/mL penicillin/streptomycin, 1 × endothelial cell growth supplement containing recombinant TSP-1 (R&D Systems, Minneapolis, MN; Cat# 3074-TH-050), Aβ_1-42_ (Nanjing Peptide Biotech Ltd. China; Cat# 107761–42-2) or CD36 blocking peptide (Fab Gennix, Frisco, TX; Cat# P-CD36) was seeded onto angiogenesis slides and incubated for 4 h at 37 °C in 5% CO_2_. Images were captured and numbers of nodes, sprouts and total tube length were measured and quantified using Image J (NIH, Bethesda, MD).

### RNA sequencing

RNA sequencing (RNA-seq) data for meningeal lymphatic endothelial cells from 6-month-old 5 × FAD male mice and WT mice were obtained from the Gene Expression Omnibus (GEO) database under the accession number GSE245658. Additional bulk RNA-seq data at three different ages (4, 8, and 18 months) in the hippocampus of 5 × FAD mice and control mice were sourced from the database with the accession number GSE168137. The count data were utilized for the quantification of gene expression. Normalization and differential expression analysis were performed using the DESeq2 package (version 1.32.0) in R (version 4.3.1). Genes with an adjusted *P*-value less than 0.05 were considered differentially expressed. Principal component analysis (PCA) and hierarchical clustering were conducted to evaluate the overall variance and sample clustering. A specific list of genes, obtained from literature screening, was analyzed to ascertain differential expression across various groups. The expression levels of these genes were extracted and normalized. The gene expression levels in the histogram were normalized using the FPKM value, subtracted by the mean and divided by the standard deviation. Differential expression analysis was conducted for each gene in the list across different groups.

### Statistical analysis

Experimenters were blinded to the identity of experimental groups from the time of euthanasia until the end of data collection. Unpaired Student's *t*-test was used to compare differences between two groups. One-way ANOVA with a Tukey *post-hoc* test was used to compare three independent groups. For comparison of multiple factors (e.g., genotype versus treatment), a two-way ANOVA with a Tukey *post-hoc* test was used. A repeated-measures two-way ANOVA with a Tukey *post-hoc* test was applied for repeated observations of multiple factors. Data are presented as mean ± SEM. Statistical analysis was conducted using R (version 4.3.1) and Prism 8.0 (GraphPad Software, Inc. Boston, MA).

## Results

### Impaired meningeal lymphatic vessels in 5 × FAD mice

**Fig. 1:**
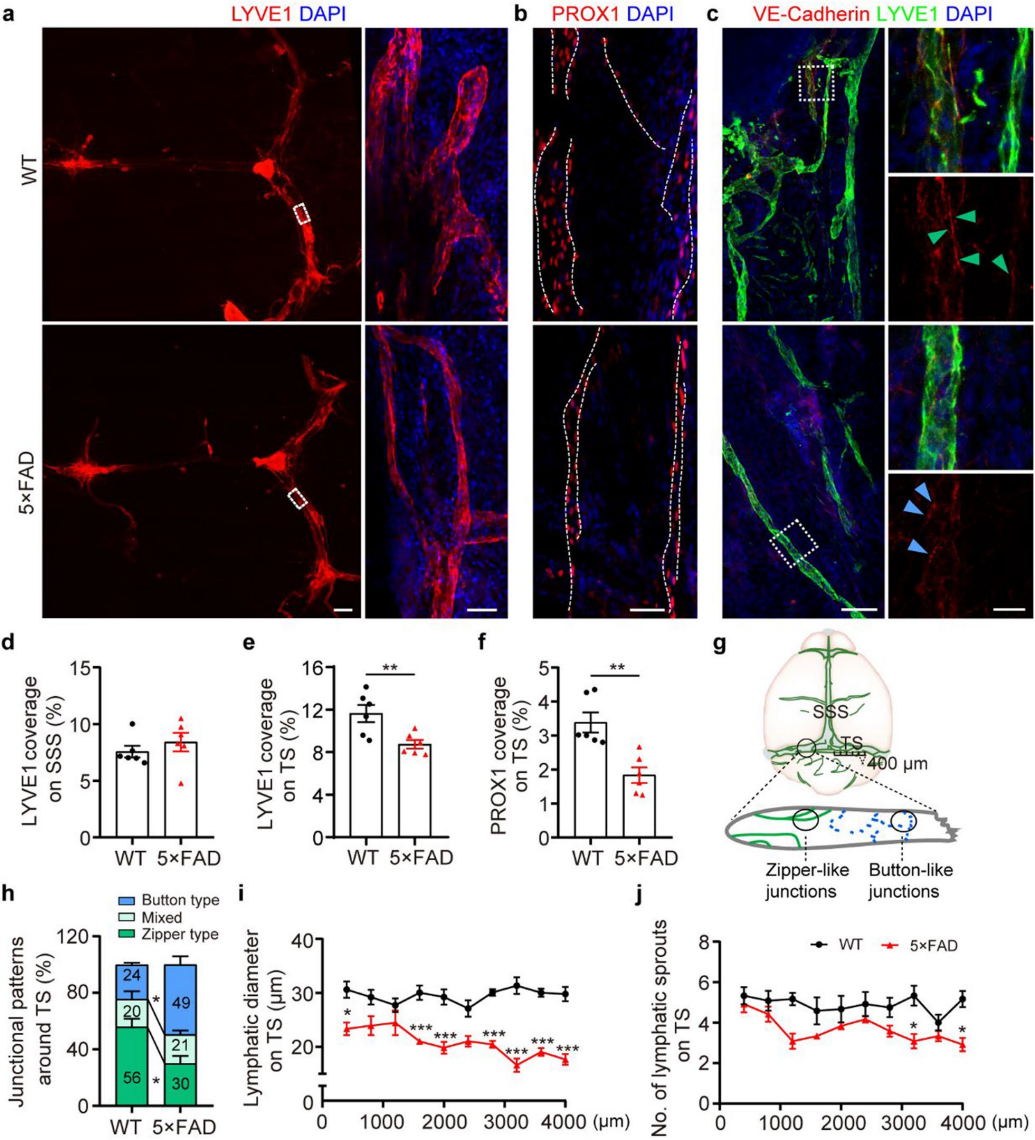
Impaired meningeal lymphatic plasticity in 5 × FAD mice. **a-c** Representative images of LYVE1^+^ (**a**), PROX1^+^ (**b**) and VE-Cadherin^+^ LYVE1^+^ (**c**) meningeal lymphatic vessels in meninges. Scale bars, 800 μm (left) and 50 μm (right) in (**a**), 50 μm in (**b**), 100 μm (left) and 20 μm (right) in (**c**). Arrowheads indicate the dominant junctional pattern. Zipper-like junctions (green arrowheads) are defined as continuous junctions at cell–cell borders of LECs, while button-like junctions (blue arrowheads) are defined as dot-like, discontinuous junctions, roughly parallel linear segments of VE-Cadherin. **d, e** Quantification of LYVE1 coverage area on the TS and superior sagittal sinus (SSS) (*n* = 6 per group). **f** Quantification of PROX1^+^ coverage area on the TS (*n* = 6 per group). **g, h** Cartoon diagram (**g**) and quantification of VE-Cadherin^+^ lymphatic vessel junctions (**h**). VE-Cadherin immunostaining at least 3.5 μm in length was recognized as zipper junctions, while button-like junctions were defined as a length of 0.5–3.2 μm and a spacing of 2.9 ± 0.3 μm. **i, j** Quantification of the diameters (**i**) and sprout numbers (**j**) of lymphatic extensions in adjacent sections of meningeal lymphatics along TS (*n* = 6 per group; bilateral TS per mouse). The lymphatics on the left and right TS were divided into 10 segments (400 μm each), respectively. Data represent the mean ± SEM; significance was evaluated with unpaired Student’s *t*-test (**d-f**) or one-way ANOVA with Tukey *post-hoc* test (**h**) or repeated-measures two-way ANOVA with Tukey *post-hoc* test (**i, j**). *P < 0.05, **P < 0.01, ***P < 0.001

The meningeal lymphatic vessels which drain brain macromolecule metabolites into the peripheral system, have been reported to be impaired in both aged and AD mice [10, 13, 14]. Consistently, we found significant decreases in the coverage of lymphatic vessels positive for lymphatic vascular endothelial hyaluronan receptor 1 (LYVE-1) and prospero homeobox protein 1 (PROX1), along the transverse sinus (TS) rather than superior sagittal sinus (SSS) of 6.5-month-old 5 × FAD mice, compared with age-matched WT mice (Fig. 1a, b, d–f). Under physiological conditions, the TS lymphatic vessels consist mostly of a zipper-like junctional pattern of LECs. Insufficient continuous zipper connections are related to impaired lymphatic flow [15, 28]. In the TS lymphatic vessels of 5 × FAD mice, there was a decrease in tight zipper-like LEC junctions and an increase in button-like LEC junctions compared to those in WT mice (Fig. 1c, g, h). Quantitative analyses also showed reductions in the diameter and the number of sprouts of TS lymphatic vessels in 5 × FAD mice, further indicating impaired lymphangiogenesis (Fig. 1i, j).

### Activated astrocytes with increased production of TSP-1 in 5 × FAD mice

**Fig. 2:**
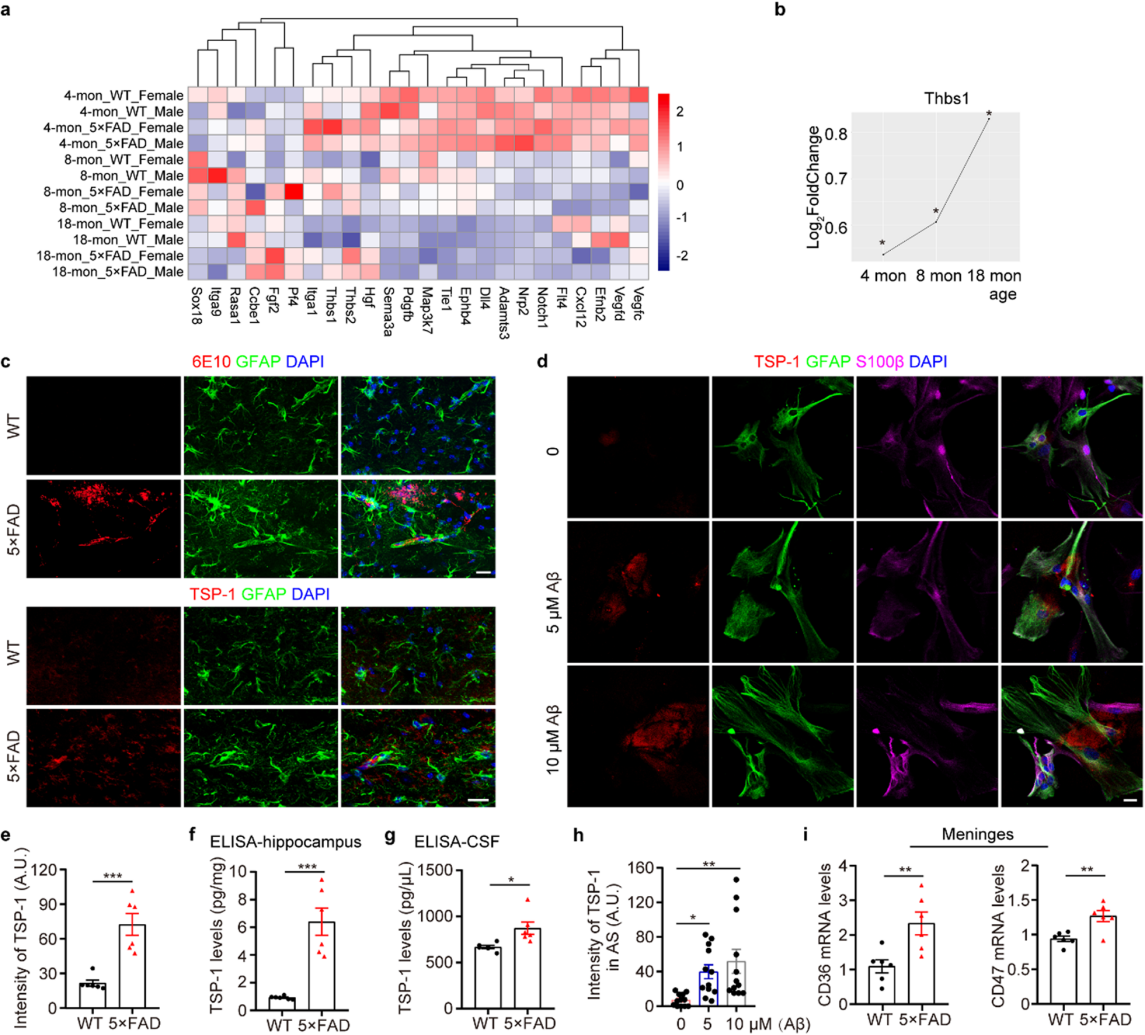
Increased TSP-1 levels in the hippocampus of 5 × FAD mice and cultured astrocytes exposed to Aβ_1-42_. **a** Heat map showing the relative expression level of genes associated with lymphangiogenesis in the hippocampus. **b** The line graph of the log2 fold change (5 × FAD vs. WT) of *Thbs1* expression in the hippocampus at three different ages. **c**,** e** Representative images of GFAP, 6E10 and TSP-1 staining and quantification of the intensity of TSP-1 in the hippocampus (*n* = 6 per group). Scale bar, 20 μm (top) and 50 μm (bottom). **d**,** h** Representative images of astrocytes (labeled with GFAP and S100β) and TSP-1 staining and quantification of the fluorescence intensity of TSP-1 in primary astrocytes (*n* = 12 per group). Scale bar, 20 μm. **f, g** ELISA assay for TSP-1 levels from the hippocampus (**f**) (*n* = 6 per group) and CSF samples (**g**) (CSF from two mice combined into one sample, 6 samples per group). **i** Relative mRNA expression of *CD36* and *CD47* in the meninges (*n* = 6 per group). Data represent the mean ± SEM; significance was evaluated with unpaired Student’s *t*-test (**e–g**,** i**) or one-way ANOVA with Tukey *post-hoc* test (**h**). *P < 0.05, **P < 0.01, ***P < 0.001

To investigate the underlying mechanism of impaired meningeal lymphangiogenesis in 5 × FAD mice, we conducted an RNA-seq analysis of LECs sorted from the meninges of 6-month-old WT and 5 × FAD mice (GSE245658). Notably, the classical factors regulating lymphangiogenesis, such as *Vegfc* and *Vegfd*, were not significantly different in meningeal LECs between WT and 5 × FAD mice (Fig. S1a-c). This suggested that the soluble cytokines regulating meningeal lymphangiogenesis are not produced by LECs but may originate from brain cells. Consequently, we further evaluated published RNA-sequence data from the hippocampus of WT and 5 × FAD mice at 4, 8, and 18 months of age (GSE168137) and screened the literature for factors that regulate lymphangiogenesis. We discovered that *Thbs1*, the gene encoding the protein TSP-1, is age-dependently elevated in the 5 × FAD group (Fig. 2a, b, Fig. S2).

TSP-1 is an endogenous inhibitor of lymphangiogenesis, which plays a crucial role in tumor metastasis and transplant outcome [29]. Notably, TSP-1 is a secreted protein primarily produced by astrocytes in the CNS, particularly up-regulated when astrocytes are activated [30, 31]. Consistently, double immunofluorescence revealed that astrocytes surrounding the plaques were activated, with increased TSP-1 expression in the hippocampus of 6.5-month-old 5 × FAD mice (Fig. 2c, e, Fig. S3). ELISA further showed high concentrations of TSP-1 in the hippocampus and CSF of 5 × FAD mice (Fig. 2f, g). AD mice also exhibited high mRNA expression of TSP-1 receptors CD36 and CD74 in the meningeal lymphatic endothelial cells, compared with WT mice (Fig. 2i). Furthermore, when treated with Aβ_1-42_ in vitro, primary astrocytes showed increased expression of TSP-1 in a dose-dependent manner (Fig. 2d, h).

### Astrocyte-specific knockdown of TSP-1 enhances meningeal lymphatic vessel plasticity and drainage in 5 × FAD mice

**Fig. 3:**
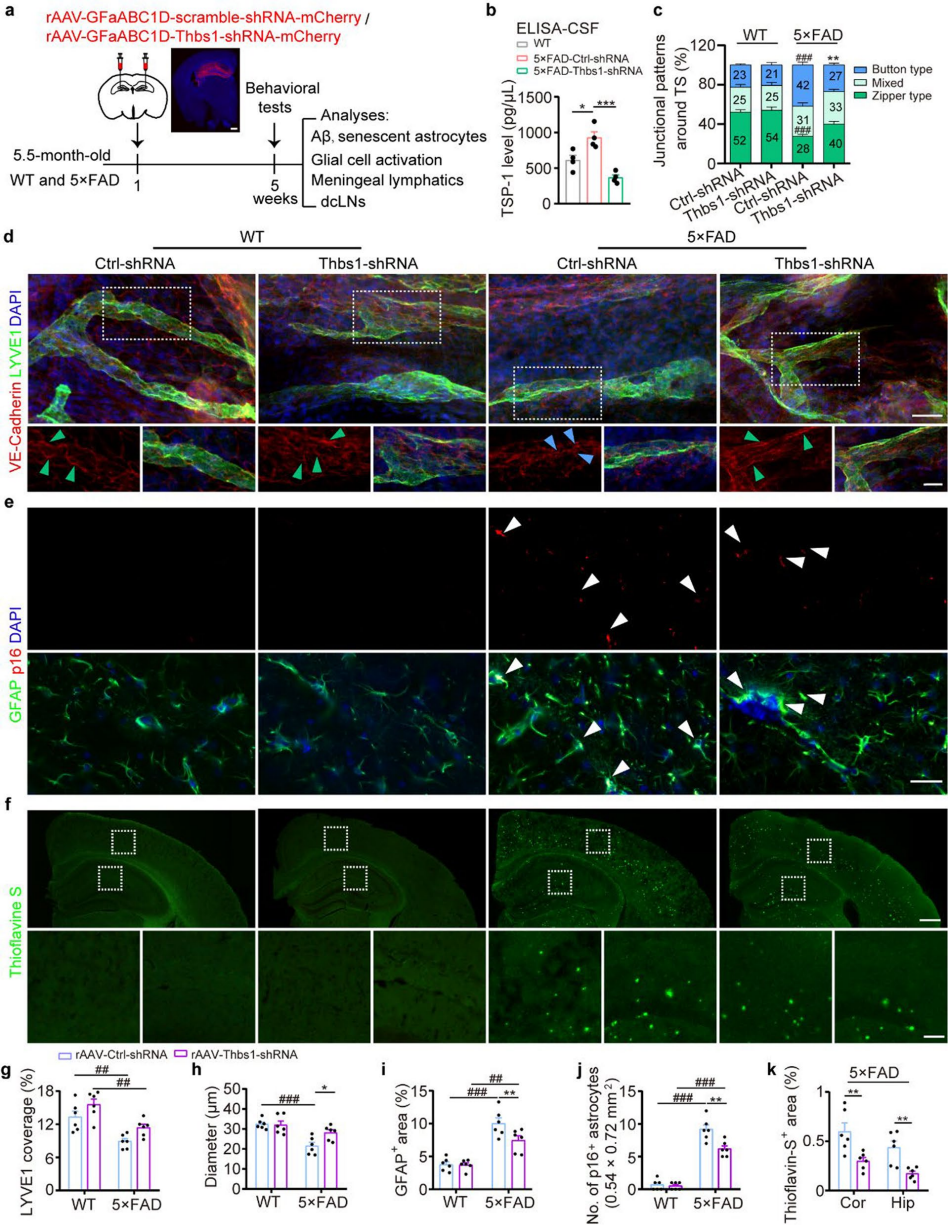
Astrocyte-specific *Thbs1* knockdown enhanced meningeal lymphatic vessel plasticity and alleviated accumulation of Aβ and senescent astrocytes in the hippocampus of 5 × FAD mice. **a** Schematic of astrocyte-specific *Thbs1* knockdown experiment. Representative images of mCherry staining showing hippocampal injection sites. Scale bar, 500 μm. **b** ELISA assay for TSP-1 levels in the CSF (CSF from two mice combined into one sample, 4 samples per group). **c, d**, **g, h** Representative images of LYVE1 and VE-Cadherin staining (**d**) and quantification of VE-Cadherin^+^ lymphatic vessel junctions (**c**), LYVE1^+^ area and diameter of LYVE1^+^ vessels (**h**) among TS region (**g**) (*n* = 6 per group). Scale bar, 40 μm (top) and 20 μm (bottom). Arrowheads indicate the dominant junctional pattern, zipper junctions (green arrowheads) and button junctions (blue arrowheads). **e**, **i, j** Representative images of GFAP^+^ senescent astrocytes (white arrowheads) characterized by high expression of p16 (**e**) and quantification of GFAP^+^ area (**i**) and p16^+^ GFAP^+^ astrocytes in the hippocampal lacunosum moleculare layer (LMol) (**j**) (*n* = 6 per group). Scale bar, 30 μm. **f**,** k** Representative images of Thioflavin-S staining and quantification of Aβ plaque areas in the hippocampus and its surrounding cortical area (*n* = 6 per group). Scale bar, 400 μm (top) and 100 μm (bottom). Data are presented as mean ± SEM; significance was evaluated with two-way ANOVA with Tukey *post-hoc* test (**c**, **g-k**, *P < 0.05, AAV-ctrl-shRNA vs AAV-Thbs1-shRNA, ^##^*P* < 0.01, ^###^*P* < 0.001, WT vs 5 × FAD) or one-way ANOVA with Tukey *post-hoc* test (**b**, *P < 0.05, **P < 0.01, ***P < 0.001)

To investigate the contribution of astrocyte-derived TSP-1 to impaired meningeal lymphatic drainage in AD, we used an AAV-Thbs1-shRNA-mCherry construct (controlled by the GfaABC1D promoter) to selectively knock down *Thbs1* in the hippocampal astrocytes of 5 × FAD mice (Fig. 3a). We confirmed reduced TSP-1 levels in the hippocampus and in the CSF of 5 × FAD mice, demonstrating the efficacy of the *Thbs1*-shRNA virus (Fig. 3b and Fig. S4a, c). Astrocyte-specific knockdown of *Thbs1* also resulted in an increased diameter of LYVE-1^+^ vessels and continuous zipper-like patterns of LECs in the meningeal tissue (Fig. 3c, d, g, h), as well as a reduction in the accumulation of Aβ plaques and senescent astrocytes, along with decreased glial activation in the brain parenchyma of 5 × FAD mice (Fig. 3e, f, i-k, Fig. S4b, d, e). Furthermore, immunofluorescence staining using monoclonal antibodies against glial fibrillary acidic protein (GFAP) and rabbit IgG antibodies specific for human Aβ to avoid recognizing endogenously produced mouse IgG1 revealed the presence of non-tissue self-produced antigens or non-specific markers in the dCLNs (Fig. S5a). This supports the notion that these macromolecules are cleared from the brain to the peripheral lymph system [32]. Elevated Aβ and GFAP signals were observed in the dCLNs of 5 × FAD mice following specific knockdown of TSP-1 in hippocampal astrocytes (Fig. S5b, c). As anticipated, the 5 × FAD mice with astrocyte-specific knockdown of TSP-1 showed increased number of entries into the novel arm during the Y-maze test, yet the performance in the NOR test was not affected (Fig. S6a-d), suggesting partial mitigation of cognitive impairment. These findings indicate that the astrocyte-specific knockdown of TSP-1 exerts a therapeutic effect on AD model mice by enhancing meningeal lymphatic plasticity and drainage.

### TSP-1 aggravates the inhibitory effects of Aβ on lymphatic vessel formation and plasticity

To confirm the involvement of TSP-1 in lymphatic vessel formation and plasticity, a tube formation assay was initially performed using SVEC4-10 cells, a cell line of LECs [33]. SVEC4-10 cells expressed high levels of lymphatic endothelial markers such as LYVE1, PROX1, and vascular endothelial growth factor receptor 3 (VEGFR3), while exhibiting low expression of vascular endothelial cell markers, including CD31, CD34, and FLI-1, compared with HAECs, a vascular endothelial cell line (Fig. S7a-d). The results indicated that treatment with 200 ng/mL TSP-1 led to reductions in the numbers of nodes and sprouts, as well as the total tube length in SVEC4-10 cells, suggesting that TSP-1 inhibits lymphangiogenesis in vitro (Fig. S8a, b, e). Additionally, previous studies have suggested that meningeal Aβ deposition may impact the LEC plasticity [13]. In the tube formation assay of SVEC4-10 cells, no significant changes were observed in lymphangiogenesis at lower concentrations of Aβ_1-42_ treatment (0, 0.5, and 2.5 μmol/L). However, the numbers of nodes and sprouts and the total tube length were notably decreased after treatment with 5 μmol/L Aβ_1-42_ (Fig. S8c, f). We further investigated whether the combination of lower concentrations of Aβ_1-42_ and TSP-1 has a synergistic inhibitory effect on lymphangiogenesis. The results demonstrated that combined treatment with 2 μmol/L Aβ_1-42_ and 100 ng/mL TSP-1 significantly suppressed tubule formation of the SVEC4-10 cells, compared to the single-factor treatment described above, which was insufficient to inhibit lymphangiogenesis (Fig. S8d, g).

**Fig. 4:**
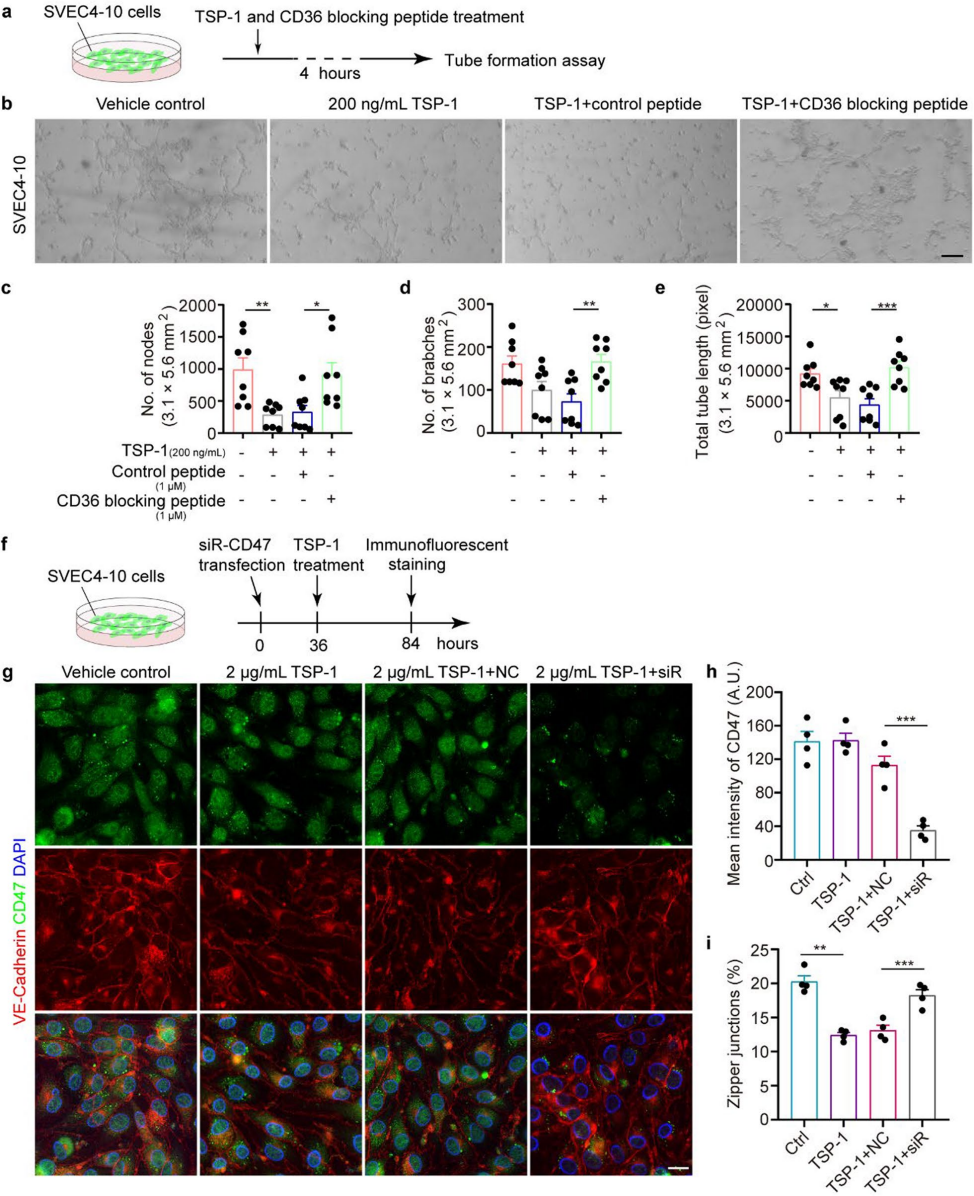
The TSP-1-CD36/CD47 signaling pathway modulates lymphangiogenesis and junction plasticity in vitro, respectively. **a** Schematic of tube formation assay. **b** Representative images of tube formation assay in the SVEC4-10 cells pre-treated with gradient concentrations of exogenous recombinant TSP-1 plus CD36 blocking peptide or control peptide. Scale bar, 100 μm. **c-e** Quantification of the numbers of nodes and sprouts as well as the total tube length in random fields (3.1 × 5.6 mm^2^), *n* = 8 per group. **f** Schematic of immunofluorescent staining experiment. SVEC4-10 cells were transfected with siRNA-CD47 for 36 h, followed by treatment with 2 μg/mL recombinant TSP-1. **g** Representative images of VE-Cadherin and CD47 staining in the SVEC4-10 cells. Scale bar, 20 μm. **h, i** Quantification of the fluorescence intensity of CD47 and the percentage of zipper-like junctions (*n* = 4 per group). Data are presented as mean ± SEM; significance was evaluated with one-way ANOVA with Tukey *post-hoc* test. *P < 0.05, **P < 0.01, ***P < 0.001

We further investigated how TSP-1 inhibits lymphatic vessel formation and plasticity. Pretreatment with a CD36-blocking peptide diminished the inhibitory effect of TSP-1 on the lymphangiogenesis of SVEC4-10 cells, as demonstrated in the tube formation assay (Fig. 4a–e). On the other hand, TSP-1 dose-dependently suppressed the VE–Cadherin zipper-like junctions (Fig. S9a, b). The inhibitory effect of TSP-1 on tube formation and VE–Cadherin continuous junctions was also confirmed in a human-derived lymphatic endothelial cell line HLEC (Fig. S9c–g). Furthermore, the inhibitory effect of TSP-1 at the concentration of 2 μg/mL on these zipper-like junctions was diminished after knockdown of CD47 in SVEC4-10 cells (Fig. S9h–j and Fig. 4f–i). In summary, these in vitro data suggest that TSP-1 inhibits lymphangiogenesis and junction plasticity through interactions with CD36 and CD47, respectively.

### Treadmill exercise reduces brain Aβ load and related pathophysiological changes

**Fig. 5:**
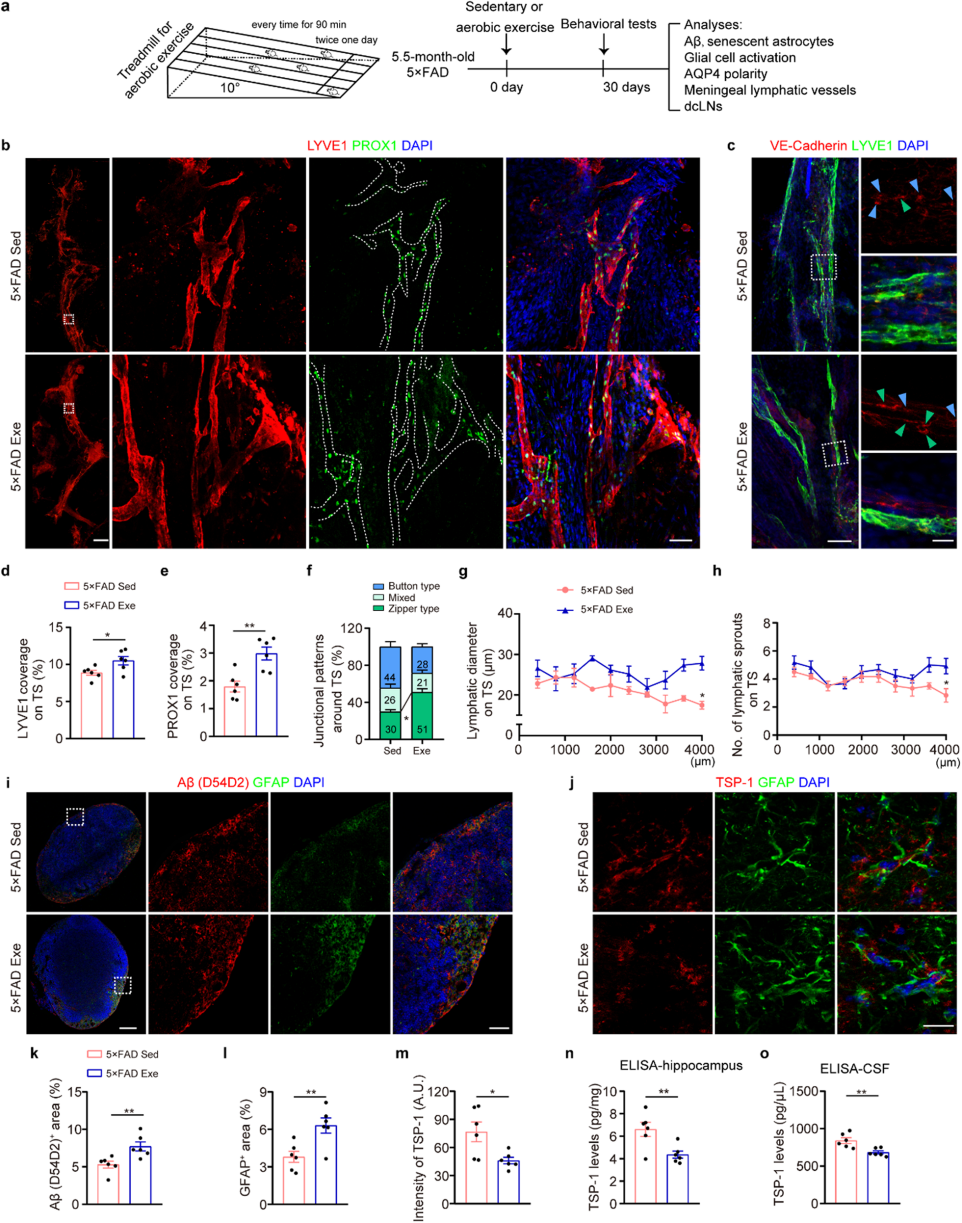
Treadmill exercise increased the plasticity of meningeal lymphatic vessels and reduced TSP-1 levels in 5 × FAD mice. **a** Schematic of treadmill training at a 10-degree incline with increasing running speeds (m/min) twice a day for 90 min for 30 days. **b, c** Representative images of PROX1^+^ LYVE1^+^ (**b**) and VE-Cadherin^**+**^ LYVE1^**+**^ (**c**) meningeal lymphatic vessels in the TS. Scale bar, 800 μm (left) and 50 μm (right) in (**b**), 100 μm (top) and 20 μm (bottom) in (**c**). Arrowheads indicate the dominant junctional pattern, zipper-like junctions (green arrowheads) and button-like junctions (blue arrowheads). **d–f** Quantification of LYVE1^+^ (**d**) and PROX1^+^ (**e**) coverage area and VE-Cadherin^+^ lymphatic vessels junctions (**f**) on the TS (*n* = 6 per group). **g, h** Quantification of the diameters (**g**) and numbers (**h**) of lymphatic extensions in adjacent sections of meningeal lymphatics along TS (*n* = 6 per group; bilateral TS per mouse). **i**, **k, l** Representative images of Aβ and GFAP staining in the dCLNs (**i**) and quantification of Aβ- (**k**) and GFAP-positive (**l**) areas in the dCLNs (*n* = 6 per group). Scale bar, 200 μm (left) and 50 μm (right). **j**,** m** Representative images of GFAP and TSP-1 staining (**j**) and quantification of the intensity of TSP-1 in the hippocampus (**m**) (*n* = 6 per group). Scale bar, 50 μm. **n, o** ELISA assay for TSP-1 levels from the hippocampus (**n**) and CSF samples (**o**) (CSF from two mice combined into one sample, 6 samples per group). Data are presented as mean ± SEM; significance was evaluated with unpaired Student’s *t*-test (**d**, **e**, **k–o**) or one-way ANOVA with Tukey *post-hoc* test (**f**) or repeated-measures two-way ANOVA with Tukey *post-hoc* test (**g, h**). *P < 0.05, **P < 0.01, ***P < 0.001

Brain Aβ deposition is a typical hallmark in AD transgenic mice [34]. We assessed whether treadmill exercise could attenuate Aβ-related brain pathology and cognitive dysfunction in 5 × FAD mice (Fig. 5a). After one month of treadmill exercise, Thioflavin-S-positive plaques, reactive gliosis, and accumulation of senescent astrocytes were significantly reduced in the hippocampus and the adjacent cortical region of 6.5-month-old 5 × FAD mice (Fig. S10a–g). Treadmill exercise also had a beneficial effect on the short-term learning and memory of 5 × FAD mice (Fig. S11a–d). However, exercise intervention did not ameliorate the anxiety-like behavior of 5 × FAD mice in the EPM test (Fig. S11e, f).

### Treadmill exercise increases meningeal lymphatic plasticity and drainage in 5 × FAD mice

We next determined whether long-term exercise-induced brain Aβ load decrease is associated with enhanced meningeal lymphatic plasticity. As expected, 6.5-month-old 5 × FAD mice that received treadmill exercise for one month showed significant increases in the area fraction, diameter, and number of sprouts of meningeal lymphatic vessels, as well as continuous lymphatic junctions (Fig. 5b–h). Furthermore, compared with the sedentary control group, the treadmill exercise group showed increased Aβ and GFAP staining in the dCLNs, indicating that exercise facilitates the drainage of brain Aβ and GFAP from the brain to the peripheral system (Fig. 5i, k, l). These results together revealed that treadmill exercise improves meningeal lymphatic vessel plasticity and drainage under AD-like pathology.

To further confirm this conclusion, in vivo two-photon imaging was used to monitor the dynamic distribution of Aβ_1-42_ at 1 h post-intrahippocampal administration in the meningeal lymphatic vessels of 6-month-old WT and 5 × FAD mice that had undergone 10 days of treadmill exercise training (Fig. S12a). As previously reported [35], Aβ_1-42_ fluorescent signals were detected in the A488-Lyve1-labeled meningeal lymphatic vessels running along the TS. Notably, a comparison of Aβ_1-42_–555 drainage through the TS regions at consecutive time points revealed a delayed clearance of the Aβ tracer in 5 × FAD mice. The treadmill exercise significantly enhanced the drainage of Aβ_1-42_–555 from the meningeal lymphatic vessels in WT mice, and there was a trend toward improvement in AD mice as well (Fig. S12b, c and Additional file Movies 1–4). Consistently, there were increased Aβ_1-42_–555 signals in the dCLNs of 5 × FAD mice after treadmill exercise (Fig. S12d, e). These results verified that exercise promotes the meningeal lymphatic drainage of Aβ from the brain to the peripheral system.

### Treadmill exercise down-regulates TSP-1 expression in astrocytes of 5 × FAD mice

We examined the expression level of TSP-1 in the hippocampus of 5 × FAD mice with or without exercise, and found that the upregulated expression of TSP-1 in GFAP-positive astrocytes was reversed by treadmill exercise (Fig. 5j and m). ELISA analysis of the hippocampus confirmed this result (Fig. 5n). ELISA analysis also indicated a significant decrease of TSP-1 level in the CSF after treadmill exercise in 5 × FAD mice (Fig. 5o). Consistently, expression of the TSP-1 receptor CD36 in meningeal lymphatic vessels was significantly increased in 5 × FAD mice compared to WT mice, but the increase was partially normalized by exercise in 5 × FAD mice (Fig. S13a, b).

Besides, the exchange of CSF and interstitial fluid mediated by AQP4 is responsible for the clearance of harmful metabolites from the brain [36]. We observed that, compared with WT littermates, the perivascular localization of astrocytic AQP4 was impaired in the hippocampus of 5 × FAD mice, a condition that was reversed by treadmill exercise (Fig. S14a–c). This suggests that treadmill exercise also facilitates AQP4-mediated glymphatic clearance of Aβ. However, the expression levels of APP and its secretases, as well as proteins involved in the transport or degradation of Aβ, were not significantly altered in the hippocampus of WT mice and 5 × FAD mice following exercise training (Fig. S15a, b). Collectively, these data suggest that long-term exercise enhances the glymphatic-meningeal lymphatic transport of Aβ, which in turn inhibits the pathological cascades of parenchymal Aβ accumulation, astrocyte activation, TSP1 secretion, and meningeal lymphatic dysfunction, thereby improving the cognitive function of 5 × FAD mice.

### EAF2 regulating TSP-1 expression in activated astrocytes exposed to Aβ

**Fig. 6:**
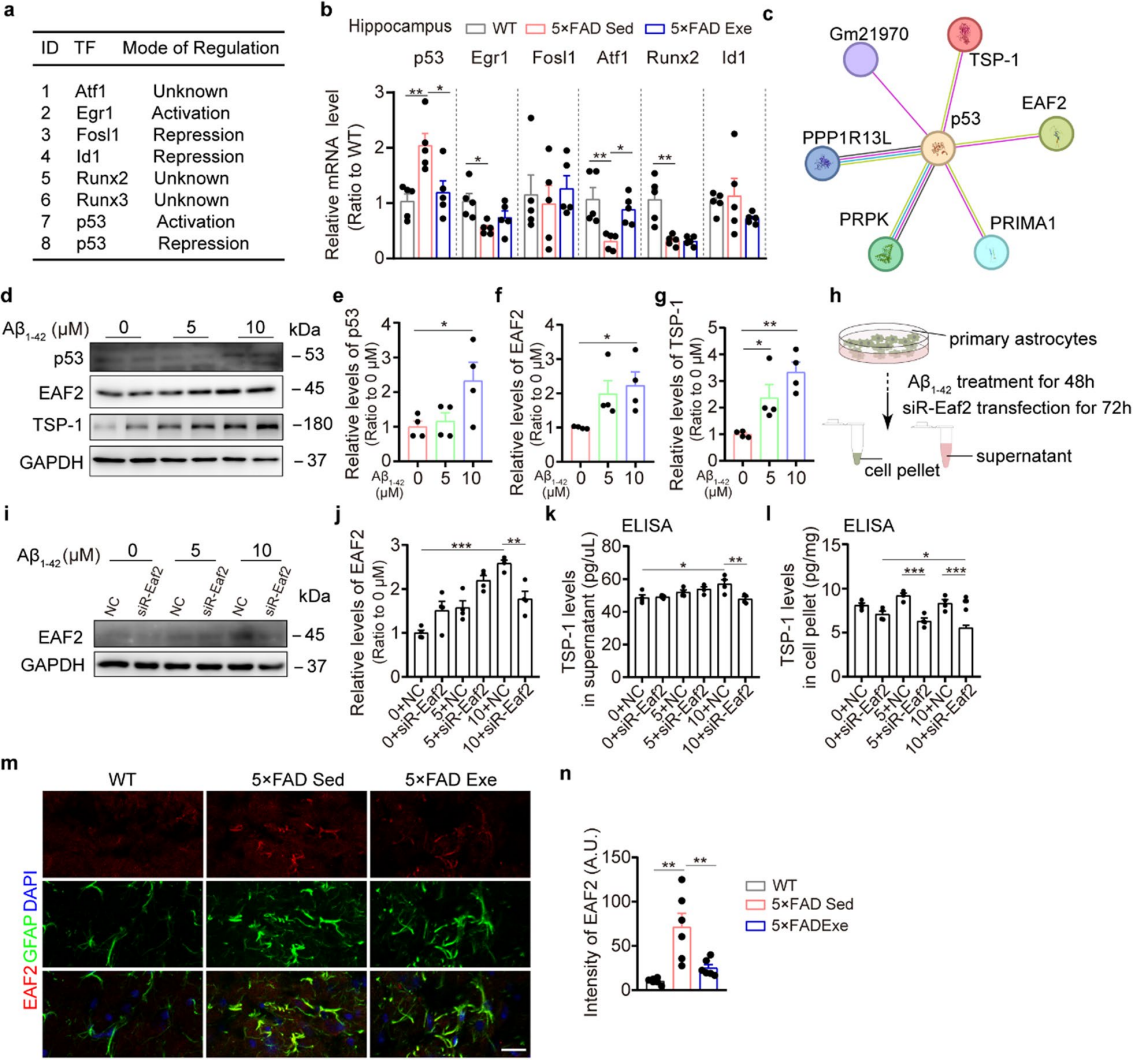
EAF2 decreased TSP-1 levels through binding to p53. **a** A list of transcription factors (TFs) that regulate *Thbs1*. **b** Relative mRNA expression of upstream regulators of TSP-1 (*p53*, *Egr1*, *Fosl1*, *Atf1*, *Runx2,* and *Id1*) in the hippocampus from 6-month-old WT and 5 × FAD mice (*n* = 5 per group). **c** Functional protein association networks among p53, EAF2 and TSP-1. **d-g** Representative Western blot bands (**d**) and densitometry analysis of p53 (**e**), EAF2 (**f**) and TSP-1 levels (**g**) in the pellet of primary astrocytes treated with gradient Aβ_1-42_ for 48 h (*n* = 4 per group). **h** Schematic of primary astrocytes treated with gradient Aβ_1-42_ for 48 h followed by knocking down of *Eaf2*. **i, j** Representative Western blot bands (**i**) and densitometry analysis (**j**) of EAF2 levels in the pellet of primary astrocytes treated with gradient Aβ_1-42_ for 48 h followed by knockdown of *Eaf2* (*n* = 4 per group). **k-l** ELISA assay for TSP-1 levels from the supernatant (**k**) and pellet (**l**) of primary astrocytes treated with gradient Aβ_1-42_ for 48 h followed by knockdown of *Eaf2* (*n* = 4 per group). **m** Representative images of EAF2 and GFAP staining and quantification of the fluorescence intensity of EAF2 in the hippocampus. Scale bar, 20 μm, *n* = 6 per group. Data represent the mean ± SEM; significance was evaluated with one-way ANOVA with Tukey *post-hoc* test. *P < 0.05, **P < 0.01, ***P < 0.001

To further explore how exercise reduces TSP-1 expression, we screened the upstream transcription factors (TFs) of TSP-1 in the hippocampus of WT mice and 5 × FAD mice following exercise training, which brought p53 into our focus (Fig. 6a, b). The regulation of TSP-1 by p53 varies across tissues and cells [37–39]. For instance, downregulation of TSP-1 has been shown to increase angiogenesis in the liver of EAF2 knockout mice. However, transfection of EAF2 alone has minimal impact on the TSP-1 promoter [40]. Functional protein association networks analysis indicated that EAF2 has a direct regulatory relationship with p53, but not with TSP-1 (Fig. 6c). Additional evidence was provided by primary astrocytes exposed to varying concentrations of Aβ_1-42_ for 48 h, which demonstrated that the levels of p53, EAF2, and TSP-1 were all elevated at a concentration of 10 μmol/L (Fig. 6d–g). Furthermore, increased TSP-1 levels in astrocyte cells and their culture medium upon exposure to Aβ_1-42_ were reversed by *Eaf2* knockdown (Fig. 6h–l). Consequently, we hypothesized that EAF2 plays a role in meningeal lymphangiogenesis by modulating TSP-1 levels through its interaction with p53. As anticipated, mRNA expression of p53 was elevated in the hippocampus of 5 × FAD mice (Fig. 6b). Additionally, EAF2 was strongly co-localized with activated astrocytes and its expression was increased in the hippocampus of 5 × FAD mice compared to WT mice (Fig. 6m, n). Collectively, these findings suggest that Aβ triggers the activation of the EAF2-p53-TSP-1 pathway in astrocytes.

### Astrocyte-specific knockdown of EAF2 improves meningeal lymphatic drainage and AD-like pathology in 5 × FAD mice

**Fig. 7:**
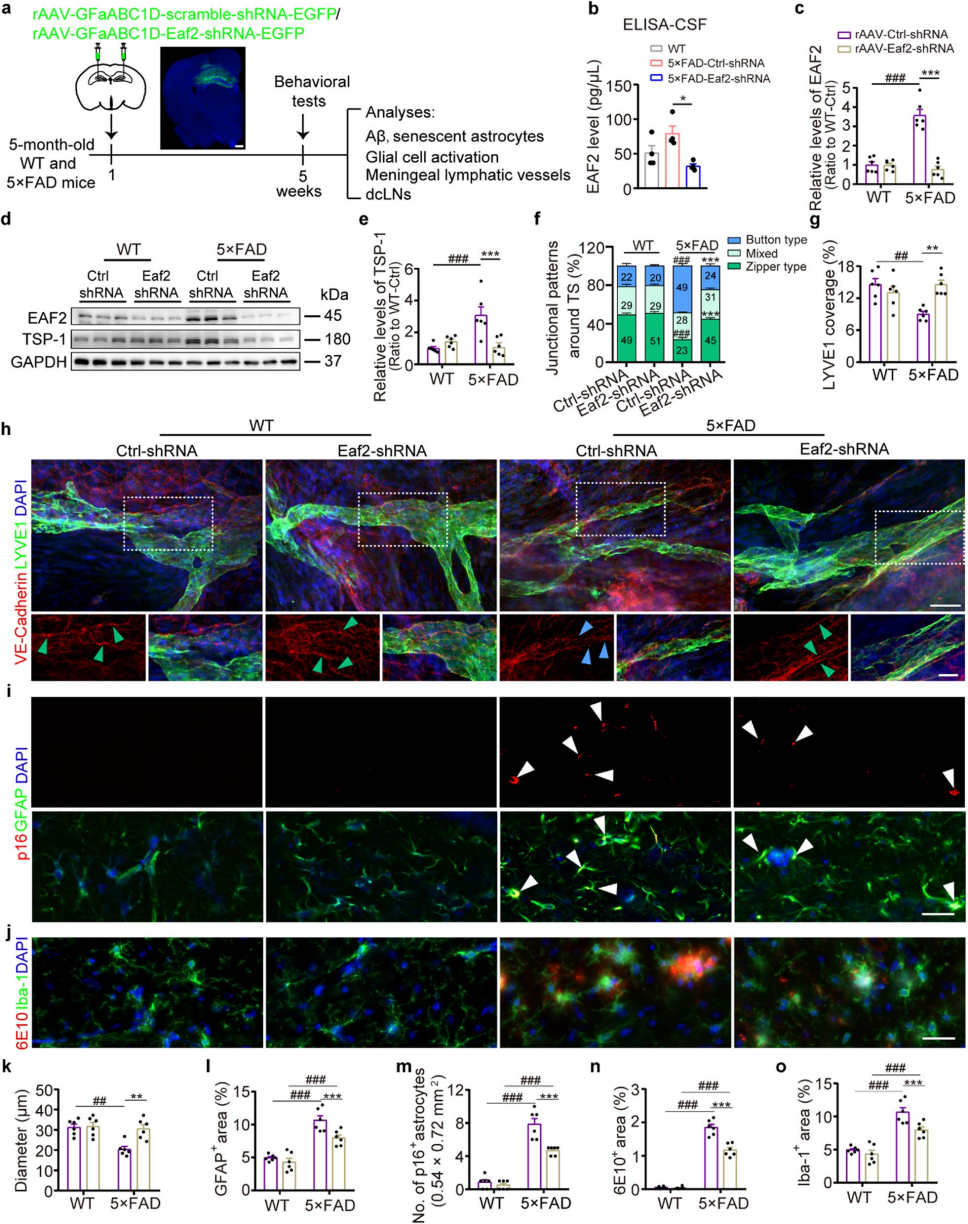
Astrocyte-specific *Eaf2* knockdown enhanced meningeal lymphatic vessel plasticity and alleviated accumulation of Aβ and senescent astrocytes in the hippocampus of 5 × FAD mice. **a** Schematic of experiments of astrocyte-specific knockdown of *Eaf2*. A representative image of EGFP staining showing hippocampal injection sites. Scale bar, 500 μm. **b** ELISA assay for EAF2 levels in the CSF (CSF from two mice combined into one sample, 4 samples per group). **c-e** Representative Western blots (**d**) and densitometry analysis of EAF2 (**c**) and TSP-1 (**e**) levels in the hippocampus (*n* = 6 per group). **f–h**, **k** Representative images of LYVE1 and VE-Cadherin staining (**h**) and quantification of VE-Cadherin^+^ lymphatic vessel junctions (**f**), the percentage of LYVE1^+^ area (**g**) and diameter of LYVE1^+^ vessels (**k**) among TS region in the meninges. *n* = 6 per group. Scale bars, 40 μm (top) and 20 μm (bottom). Arrowheads indicate the dominant junctional pattern, zipper junctions (green arrowheads) and button junctions (blue arrowheads). **i** Representative images of GFAP^+^ senescent astrocytes (white arrowheads) characterized by high expression of p16 in the hippocampal lacunosum moleculare layer (LMol). Scale bar, 30 μm. **j** Representative images of 6E10 and Iba-1 staining in the hippocampus. Scale bar, 30 μm. **l, m** Quantification of GFAP^+^ area and GFAP^+^ p16^+^ astrocytes in the LMol (*n* = 6 per group). **n, o** Quantification of the percentages of 6E10^+^ and Iba-1^+^ areas in the hippocampus, respectively (*n* = 6 per group). Data are presented as mean ± SEM; significance was evaluated with two-way ANOVA with Tukey *post-hoc* test (**c**, **e**, **f**, **g**, **k–o**, *P < 0.05, **P < 0.01, ***P < 0.001, AAV-ctrl-shRNA vs AAV-Thbs1-shRNA, ^##^*P* < 0.01, ^###^*P* < 0.001, WT vs 5 × FAD) or one-way ANOVA with Tukey *post-hoc* test (**b**, *P < 0.05, **P < 0.01, ***P < 0.001)

We next investigated whether the drainage of meningeal lymphatic vessels could be enhanced through selective knockdown of astrocytic EAF2. Five-month-old 5 × FAD mice were administered with an AAV-Eaf2-shRNA-EGFP construct (controlled by the GfaABC1D promoter) to selectively knock down *Eaf2* in hippocampal astrocytes (Fig. 7a and Fig. S16a, b). Four weeks later, EAF2 levels were also significantly decreased in the CSF of 5 × FAD mice (Fig. 7b). These 5 × FAD mice, with selective knockdown of the *Eaf2* gene in astrocytes, exhibited improved behavioral performance in the NOR test and Y-maze test (Fig. S17a–d). Furthermore, the hippocampal level of TSP-1 decreased after *Eaf2* knockdown in astrocytes of 5 × FAD mice (Fig. 7c–e). Additionally, down-regulating EAF2 in astrocytes significantly increased the coverage and diameter of LYVE1^+^ lymphatic vessels and the continuity of the zipper-like patterns of meningeal LECs (Fig. 7f, h and k). This transformation of LEC junction patterns may facilitate Aβ drainage, as evidenced by reductions in Aβ plaques, reactive gliosis, and astrocyte senescence in the hippocampus (Fig. 7i, j, l–o). In line with these findings, Aβ and GFAP levels were higher in the dCLNs compared to control groups (Fig. S18a–c). Collectively, these outcomes suggest that EAF2, derived from activated astrocytes, plays an inhibitory role in meningeal lymphangiogenesis under AD-like pathology.

## Discussion

In aged or AD mice, impaired meningeal lymphatic drainage is accompanied by decreased macromolecule draining into lymph nodes, as well as cognitive decline [13, 15]. Considering that VEGFC is essential for normal meningeal lymphatic vessel development, overexpression of VEGFC can induce meningeal lymphangiogenesis [16]. Consistently, our previous data also showed a significant increase in the drainage of GFAP into the dCLNs in aged mice after AAV1-CMV-mVEGFC treatment [10]. In the current study, we observed no alterations in the expression of VEGFC in the meninges, but significant upregulation of TSP-1, a secreted protein associated with lymphangiogenesis, in the hippocampus of 6.5-month-old AD mice, compared with WT mice.

The identification of clinical biomarkers for meningeal lymphatic injury is essential for early detection and monitoring of neurodegenerative diseases. Advanced neuroimaging techniques, such as dynamic contrast-enhanced magnetic resonance imaging (MRI) and positron emission tomography, could provide valuable insights into the changes in CSF flow and meningeal lymphatic function [15, 41, 42]. Additionally, CSF biomarkers, encompassing proteins such as Aβ, tau, and GFAP, as well as lymphatic markers such as podoplanin and LYVE-1, may indicate impaired lymphatic drainage [11, 43]. Beyond these markers, neuroinflammatory cytokines and matrix metalloproteinases, which play roles in blood–brain barrier disruption and lymphatic vessel remodeling, could offer additional evidence of lymphatic dysfunction [44, 45]. Collectively, these biomarkers could improve our capacity to detect and monitor meningeal lymphatic injury in clinical settings. However, this hypothesis requires more evidence. Previous studies have shown that TSP-1 plays a role in peripheral tumor lymphatic metastasis and corneal lymphatic vessel remodeling [29, 46]. However, the expression level of TSP-1 in CNS lymphangiogenesis has not been investigated before. Buee et al. (1992) reported that the distribution of TSPs in the brains of AD patients was comparable to that in control subjects [47]. In contrast, Son et al. (2015) found downregulation of TSP-1 in cortical samples from individuals with AD [48]. This discrepancy might be attributed to the heterogeneity of astrocyte distribution and the variable disease processes involved in AD.

TSP-1 also acts as a promoter of aging and age-associated diseases. Accumulation of TSP-1 in the extracellular matrix is frequently observed in age-related diseases [49]. In this study, we found that treadmill exercise reduced the expression level of TSP-1 in the brain parenchyma of AD mice. Similarly, RNA sequencing analysis (referring to GSE164401) also revealed a trend of decrease of hippocampal transcriptome level of *Thbs1* in mice injected with plasma from exercising mice, compared with mice receiving control plasma injection. Altogether, these data highlight that exercise interventions alleviate AD pathology by increasing meningeal lymphangiogenesis via TSP-1.

The TSP-1 receptor CD36 serves as a negative regulator of angiogenesis and lymphangiogenesis [50]. In the current study, we further demonstrated that TSP-1 can effectively suppress the proliferation of meningeal lymphatic vessels in vitro by binding to the CD36 receptor on LECs. CD36 has been implicated in maintaining lymphatic vessel integrity, which is associated with obesity and type 2 diabetes models [51]. Deletion of CD36 protects cerebral arteries from the harmful effects of Aβ_1-40_, thereby enhancing the cognitive performance of AD model mice [52]. CD47 is another receptor for TSP-1. The TSP-1–CD47 interaction modulates apoptosis of meningeal lymphatic endothelial cells in a subarachnoid hemorrhage model [53]. Elevated levels of TSP-1 impede lymphangiogenesis by activating CD47 in aortic LECs of a mouse model of atherosclerosis [54]. Our findings further indicate that TSP-1–CD47 regulates the junctional pattern of meningeal lymphatic vessels.

EAF2 is preferentially expressed in the CNS during mouse embryonic development [55]. Overexpression of EAF2 induces apoptosis and inhibits cell growth in several peripheral studies [56, 57]. However, its role in the CNS has been scarcely reported. Here we showed that EAF2 expression was upregulated in activated astrocytes of 5 × FAD mice, while exercise reversed this increase and down-regulated its binding partner p53 in 5 × FAD mice, thereby promoting the inhibition of TSP-1 by the EAF2-p53 complex. We also demonstrated that knocking down EAF2 in specific astrocytes reduced TSP-1 expression in the brain, which in turn improved meningeal lymphatic vessel function in AD mice. Collectively, these findings underscore that the EAF2–p53–TSP-1 pathway plays a crucial role in regulating meningeal lymphatic plasticity.

Epidemiological studies have reported that treadmill exercise is a generally applicable form of physical therapy to delay the aging process and improve cognitive function of patients with AD or mild cognitive impairment [58, 59]. However, there is also literature of a different view [60, 61]. Several studies have indicated that treadmill exercise has the potential to reduce Aβ load in both AD patients and transgenic AD mice [62, 63]. Here, we confirmed that treadmill exercise decreased the parenchymal Aβ deposition in 6.5-month-old 5 × FAD mice. Aβ aggregation and deposition accelerate neuroinflammation and also trigger cellular senescence [3]. Particularly, astrocytes are susceptible to senescence within the CNS [4]. Elimination of senescent glial cells using pharmacological or genetic approaches preserves cognitive function in tau transgenic mouse [5]. In this study, we demonstrated that the enhancement of learning and memory in AD mice through treadmill exercise was accompanied by increased removal of senescent astrocytes from the hippocampus. Previous studies have reported that targeting the clearance of senescent cells, such as cardiomyocytes, pancreatic β cells, and osteocytes, alleviates disease symptoms and slows the aging process [64–66]. Our findings further suggest that elimination of Aβ and senescent astrocytes may mediate the benefits of treadmill exercise against AD pathology.

A previous study reported that the glymphatic influx in the putamen, as well as the size and flow of meningeal lymphatics, are significantly increased in volunteers after long-term (12 weeks) cycling exercise as assessed by noninvasive MRI [67]. This suggests that sustained physical exercise promotes the flow of putative glymphatic and meningeal lymphatic vessels, thereby enhancing the clearance of brain metabolites in humans. Furthermore, there was no significant difference in lymphatic and meningeal lymphatic vessel flow before and after a single exercise session. Studies in mice have shown increased CSF influx after 5 weeks of voluntary running-wheel exercise [25]. Similarly, human studies have demonstrated increased middle cerebral artery compliance in volunteers engaged in moderate-to-vigorous recreational aerobic exercise [68]. Based on these findings, it is hypothesized that moderate to high intensity of exercise may significantly contribute to CSF flow and clearance of metabolites from the brain.

Growing evidence suggests that the glymphatic system acts as a functional pathway for the removal of metabolic waste from the brain parenchyma [36]. Voluntary exercise in young mice or aged mice enhances glymphatic function, leading to pro-cognitive effects [24, 25]. Furthermore, our previous study indicated that the improvement of cognitive deficits in APP/PS1 mice through voluntary exercise is dependent on the polarity of astrocytic AQP4 [26]. In the present study, we confirmed that treadmill exercise significantly reduced the activation of astrocytes and improved AQP4 polarity in the hippocampus of 5 × FAD mice. These findings suggest that the glymphatic system, which is responsible for the clearance of Aβ, could be an important target for the preventive and therapeutic effects of treadmill exercise on AD.

Furthermore, the meningeal lymphatic system serves as a crucial pathway for the elimination of metabolites and brain antigens from the CSF. Studies have demonstrated that improving the meningeal lymphatic function can improve learning and memory in both aged and AD model mice [13, 14]. Conversely, inhibiting lymphatic drainage in aged mice worsens the accumulation of perivascular senescent astrocytes within the brain parenchyma and exacerbates cognitive behavioral deficits [10]. Additionally, previous research has shown that the cerebral blood flow and CSF flow dynamics significantly increase during exercise in both humans and rodents [25, 69]. In our study, treadmill exercise increased the diameter and the number of sprouts and the continuity of VE–Cadherin junctions in the meningeal lymphatic vessels of AD mice, thereby enhancing their drainage function. This approach could potentially be a promising strategy to improve the clearance of Aβ, potentially slowing the progression of AD.

It is important to note that the treadmill exercise training was conducted at two time points a day (09:00 and 20:00) to avoid potential effects of circadian rhythms on running. We did not ascertain which time point for treadmill exercise would yield the greatest benefit for the mice. Human studies have indicated that exercise in the morning and the evening have distinct effects on the skeletal muscle molecular clock and nocturnal sleep [70, 71]. The circadian rhythm of the effect of treadmill exercise on the meningeal lymphatic vessels in humans is worthy of further clarification and will maximize the benefits of treadmill exercise, particularly for the elderly or individuals with AD.

## Conclusions

**Fig. 8:**
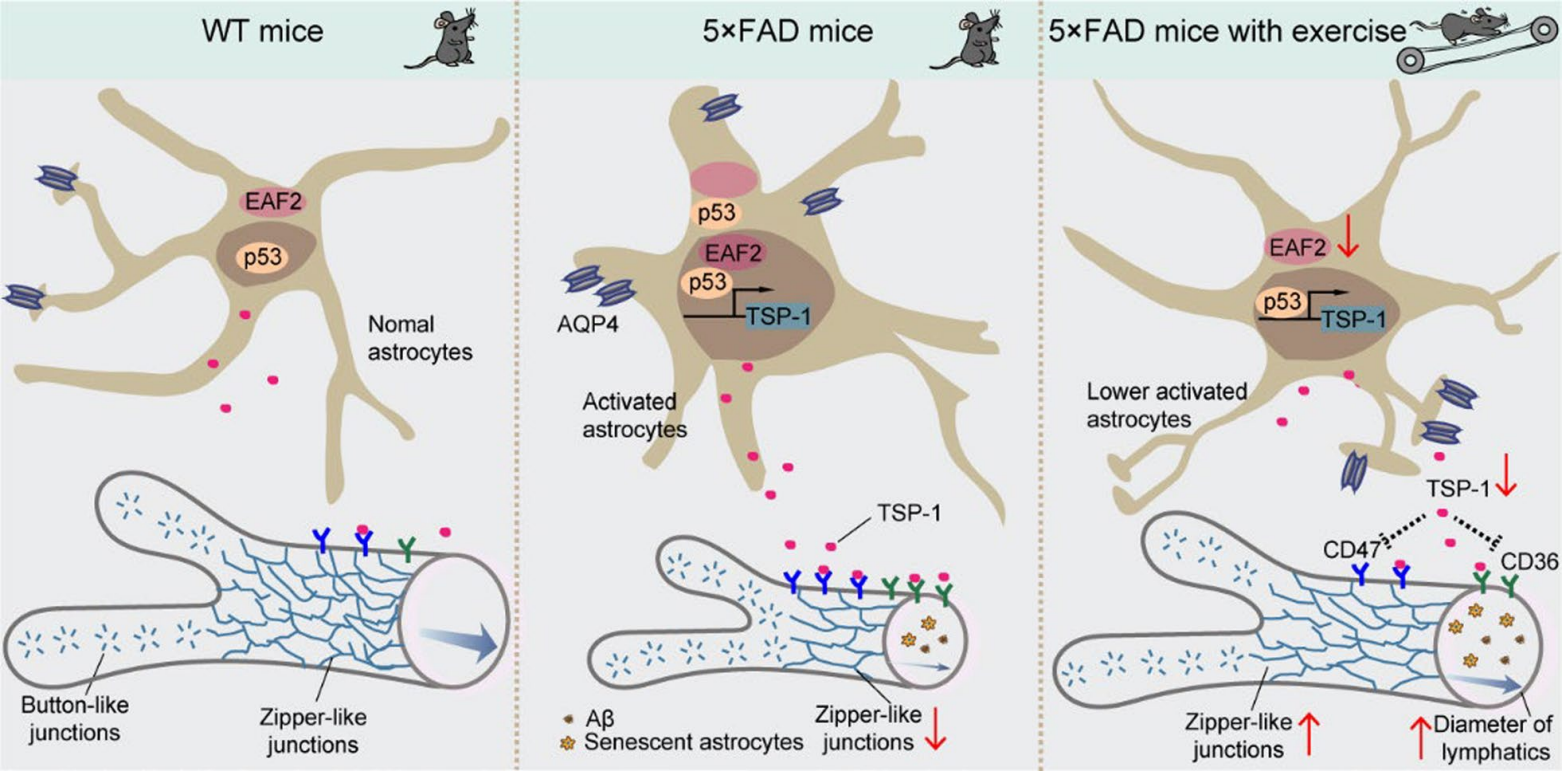
Illustration of impaired lymphangiogenesis in the AD mouse model, which can be improved by long-term exercise. Exercise increases the zipper-like junctions and the diameter of meningeal lymphatics via down-regulation of the EAF2–p53–TSP-1 pathway, which in turn facilitates drainage of brain Aβ and reduces astrocyte activation and senescence

The plasticity of meningeal lymphatics has been identified as a significant target for the drainage of metabolites from the brain. Here we demonstrate that TSP-1 produced by activated astrocytes plays a crucial role in the impairment of meningeal lymphangiogenesis in 5 × FAD transgenic mice. Astrocyte-specific knockdown of *Thbs1* or *Eaf2* promotes functional meningeal lymphatic vessel plasticity and mitigates AD-like pathology. Our results also indicate that exercise modulates the lymphangiogenesis and junctional patterns of meningeal lymphatic vessels by down-regulating the reactive astrocyte-related EAF2–p53–TSP-1 pathway (Fig. 8). Our findings suggest a novel mechanism by which exercise improves meningeal lymphatic vessel plasticity, thereby alleviating AD-related pathology.

## Supplementary Information

**Additional file 1**: **Figure S1** Expression of classical factors regulating lymphangiogenesis in lymphatic endothelial cells of WT and 5×FAD mice. **Figure S2** Age-dependent changes in factors associated with lymphangiogenesis in the hippocampus among WT and 5×FAD mice. **Figure S3** TSP1 staining in mouse brain regions of WT and 5×FAD mice. **Figure S4** Analysis of microglia and plaques after astrocyte-specific Thbs1 knockdown in the hippocampus of 5×FAD mice. **Figure S5** Astrocyte-specific Thbs1 knockdown increased GFAP and Aβ levels in the dCLNs of 5×FAD mice. **Figure S6** Astrocyte-specific Thbs1 knockdown alleviated cognitive impairment of 5×FAD mice. **Figure S7** Comparative identification of lymphatic endothelial cell lines of SVEC4-10 and vascular endothelial cell lines of HAECs. **Figure S8** The inhibitory of TSP-1 and Aβ on lymphatic vessel formation and plasticity via SVEC4-10 cells in vitro. **Figure S9** TSP-1 dose-dependently inhibited VE-Cadherin-formed zipper-like junctions in vitro. **Figure S10** Treadmill exercise alleviated deposition of Aβ, reactive microglosis and astrocyte senescence of 5×FAD mice. **Figure S11** Treadmill exercise alleviated cognitive deficits of 6.5-month-old 5×FAD mice. **Figure S12** Treadmill exercise enhanced meningeal lymphatic vessels function to drain Aβ of 6.5-month-old 5×FAD mice. **Figure S13** Treadmill exercise down-regulated the elevated CD36 levels in the meninges of 6.5-month-old 5×FAD mice. **Figure S14** Treadmill exercise improved perivascular AQP4 localization of 6.5-month-old 5×FAD mice. **Figure S15** Analysis of treadmill exercise on Aβ production and clearance-related enzyme of 6.5-month-old 5×FAD mice. **Figure S16** Astrocyte-specific Eaf2 knockdown in the hippocampus of 5×FAD mice. **Figure S17** Astrocyte-specific Eaf2 knockdown alleviated cognitive impairment of 5×FAD mice. **Figure S18** Astrocyte-specific Eaf2 knockdown increased GFAP and Aβ levels in the dCLNs of 5×FAD mice

**Additional file 2**: Original western blot bands

**Additional file 3**: Movie 1. 3D projections of the meningeal lymphatic vessels and Aβ1-42-555 of WT sedentary mice by two-photon imaging. Red indicates Aβ1-42 and green indicates LYVE-1 in the movies

**Additional file 4**: Movie 2. 3D projections of the meningeal lymphatic vessels and Aβ1-42-555 of WT exercised mice by two-photon imaging. Red indicates Aβ1-42 and green indicates LYVE-1 in the movies

**Additional file 5**: Movie 3. 3D projections of the meningeal lymphatic vessels and Aβ1-42-555 of 5×FAD sedentary mice by two-photon imaging. Red indicates Aβ1-42 and green indicates LYVE-1 in the movies

**Additional file 6**: Movie 4. 3D projections of the meningeal lymphatic vessels and Aβ1-42-555 of 5×FAD exercised mice by two-photon imaging. Red indicates Aβ1-42 and green indicates LYVE-1 in the movies

## Data Availability

All supporting information and data are available in the article and supplementary files.

## Abbreviations

AAV: Adeno-associated virus
AD: Alzheimer’s disease
AQP4: Aquaporin 4
Aβ: Amyloid-β
CNS: Central nervous system
CSF: Cerebrospinal fluid
DAPI: 4',6-Diamidino-2-phenylindole dihydrochloride
dCLNs: Deep cervical lymph nodes
EAF2: Eleven nineteen lysine rich leukemia -associated factor 2
ELISA: Enzyme-linked immunosorbent assay
EPM: Elevated plus maze
GFAP: Glial fibrillary acidic protein
Iba-1: Ionized calcium-binding adaptor molecule 1
LECs: Lymphatic endothelial cells
LYVE-1: Lymphatic vascular endothelial hyaluronan receptor 1
NOR: Novel object recognition
PROX1: Prospero homeobox protein 1
TFs: Transcription factors
TS: Transverse sinus
TSP-1: Thrombospondin-1
VEGFC: Vascular endothelial growth factor C
