# Supplementary material for: Long-term exercise enhances meningeal lymphatic vessel plasticity and drainage in a mouse model of Alzheimer's disease
Source: Transl Neurodegener. 2025 Jul 25;14:37. doi: 10.1186/s40035-025-00497-2 (PMC12291319; doi:10.1186/s40035-025-00497-2)
Supplement: Supplementary file 1 — Additional file 1: Figure S1 Expression of classical factors regulating lymphangiogenesis in lymphatic endothelial cells of WT and 5×FAD mice. Figure S2 Age-dependent changes in factors associated with lymphangiogenesis in the hippocampus among WT and 5×FAD mice. Figure S3 TSP1 staining in mouse brain regions of WT and 5×FAD mice. Figure S4 Analysis of microglia and plaques after astrocyte-specific Thbs1 knockdown in the hippocampus of 5×FAD mice. Figure S5 Astrocyte-specific Thbs1 knockdown increased GFAP and Aβ levels in the dCLNs of 5×FAD mice. Figure S6 Astrocyte-specific Thbs1 knockdown alleviated cognitive impairment of 5×FAD mice. Figure S7 Comparative identification of lymphatic endothelial cell lines of SVEC4-10 and vascular endothelial cell lines of HAECs. Figure S8 The inhibitory of TSP-1 and Aβ on lymphatic vessel formation and plasticity via SVEC4-10 cells in vitro. Figure S9 TSP-1 dose-dependently inhibited VE-Cadherin-formed zipper-like junctions in vitro. Figure S10 Treadmill exercise alleviated deposition of Aβ, reactive microglosis and astrocyte senescence of 5×FAD mice. Figure S11 Treadmill exercise alleviated cognitive deficits of 6.5-month-old 5×FAD mice. Figure S12 Treadmill exercise enhanced meningeal lymphatic vessels function to drain Aβ of 6.5-month-old 5×FAD mice. Figure S13 Treadmill exercise down-regulated the elevated CD36 levels in the meninges of 6.5-month-old 5×FAD mice. Figure S14 Treadmill exercise improved perivascular AQP4 localization of 6.5-month-old 5×FAD mice. Figure S15 Analysis of treadmill exercise on Aβ production and clearance-related enzyme of 6.5-month-old 5×FAD mice. Figure S16 Astrocyte-specific Eaf2 knockdown in the hippocampus of 5×FAD mice. Figure S17 Astrocyte-specific Eaf2 knockdown alleviated cognitive impairment of 5×FAD mice. Figure S18 Astrocyte-specific Eaf2 knockdown increased GFAP and Aβ levels in the dCLNs of 5×FAD mice [file 40035_2025_497_MOESM1_ESM.docx]

**Supplemental information**

**Long-term exercise enhances meningeal lymphatic vessel plasticity and drainage in a mouse model of Alzheimer's disease**

**Contents**

Supplementary Figures 1-18,

Supplementary Tables 1-2.

**
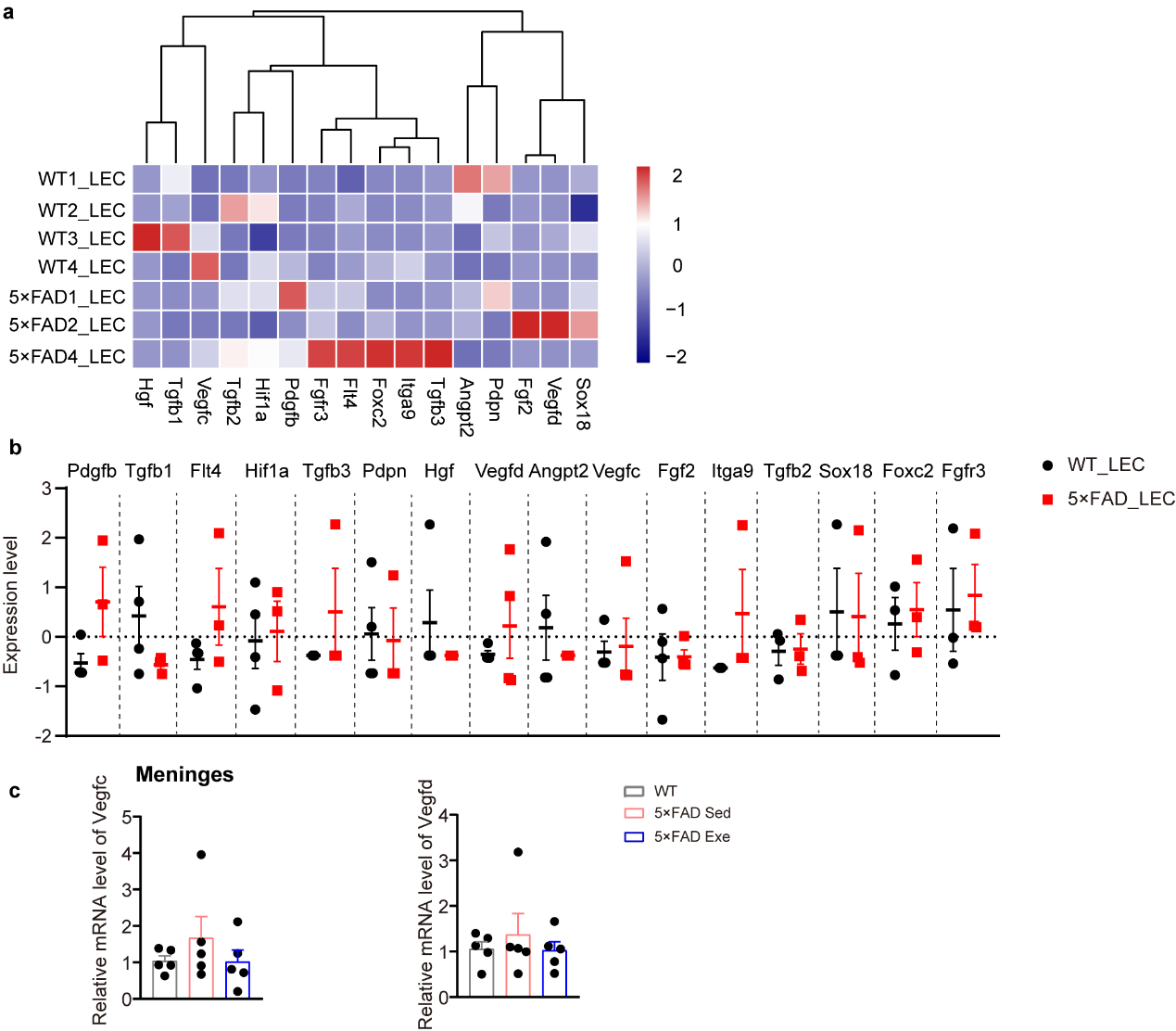
Figure S1** Expression of classical factors regulating lymphangiogenesis in lymphatic endothelial cells of WT and 5×FAD mice. **a** Heat map showing the relative expression level of genes associated with lymphangiogenesis in lymphatic endothelial cells. **b** The graph of *Vegfc, Vegfd, Flt4, Fgfr3, Pdpn, Angpt1, Angpt2, Foxc2, Hoxd8, Fgf2, Pdgfb, Hgf, Hif1a, Cc121, Tgfb1, Tgfb2, Tgfb3, ltga9,* and *Sox18* expression in lymphatic endothelial cells. **c** Relative mRNA levels of *Vegfc* and *Vegfd* in meninges (n = 5 per group). Data represent the mean ± SEM; significance was evaluated with one-way ANOVA with Tukey post hoc test (**c**).


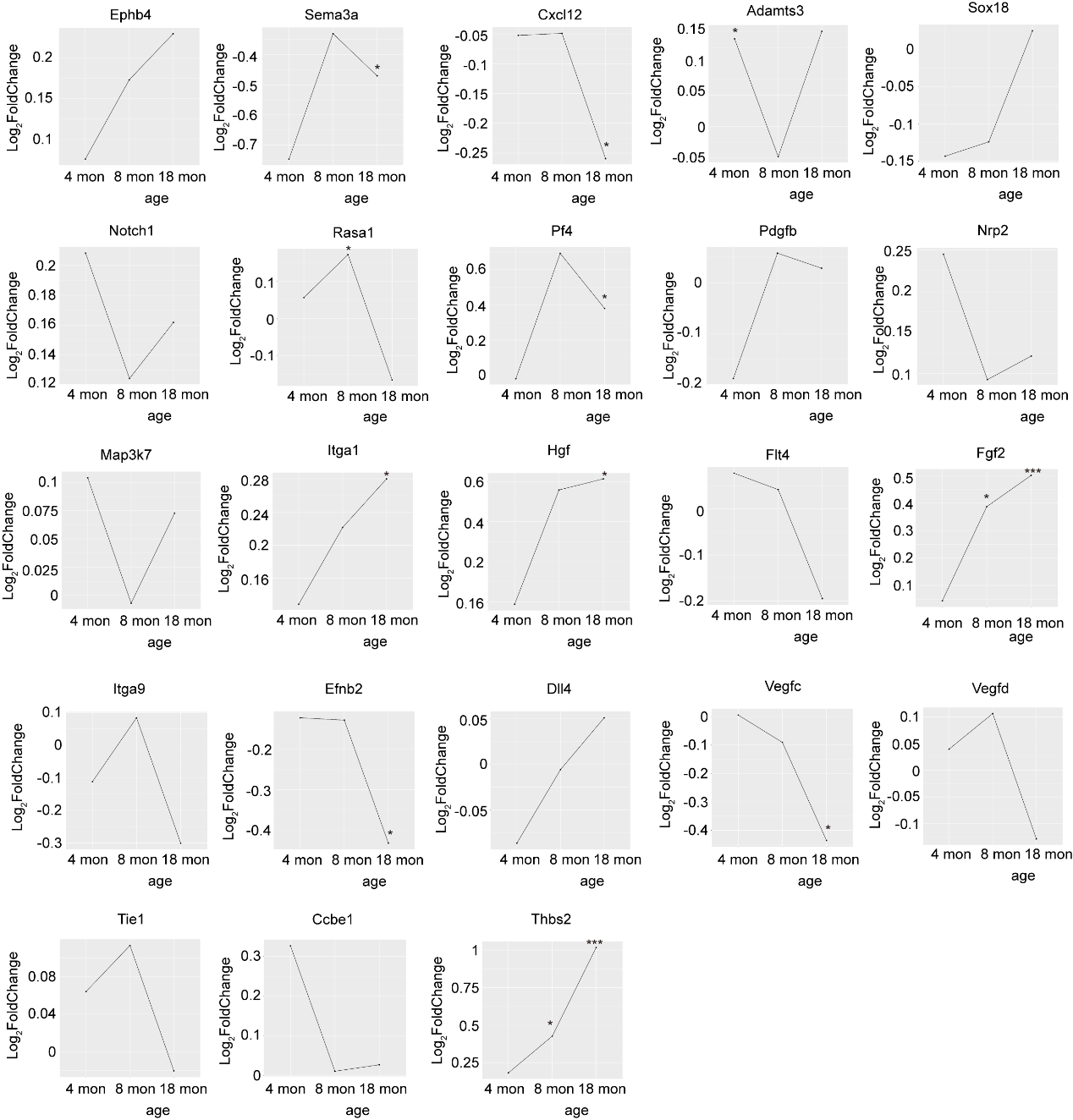


**Figure S2** Age-dependent changes in factors associated with lymphangiogenesis in the hippocampus among WT and 5×FAD mice. The line graph of the log2 fold change (5×FAD vs. WT) of *Vegfc*, *Vegfd*, *Efnb2*, *Cxcl12*, *FIt4*, *Notch1*, *Nrp2*, *Adamts3*, *Dll4*, *Ephb4*, *Tie1*, *Map3k7*, *Pdgfb*, *Sema3a*, *Hgf*, *Thbs2*, *Itga1*, *Pf4*, *Fgf2*, *Ccbe1*, *Rasa1*, *It*ga9, and *Sox18* expression in the hippocampus at three different ages (*p < 0.05, ***p < 0.001).


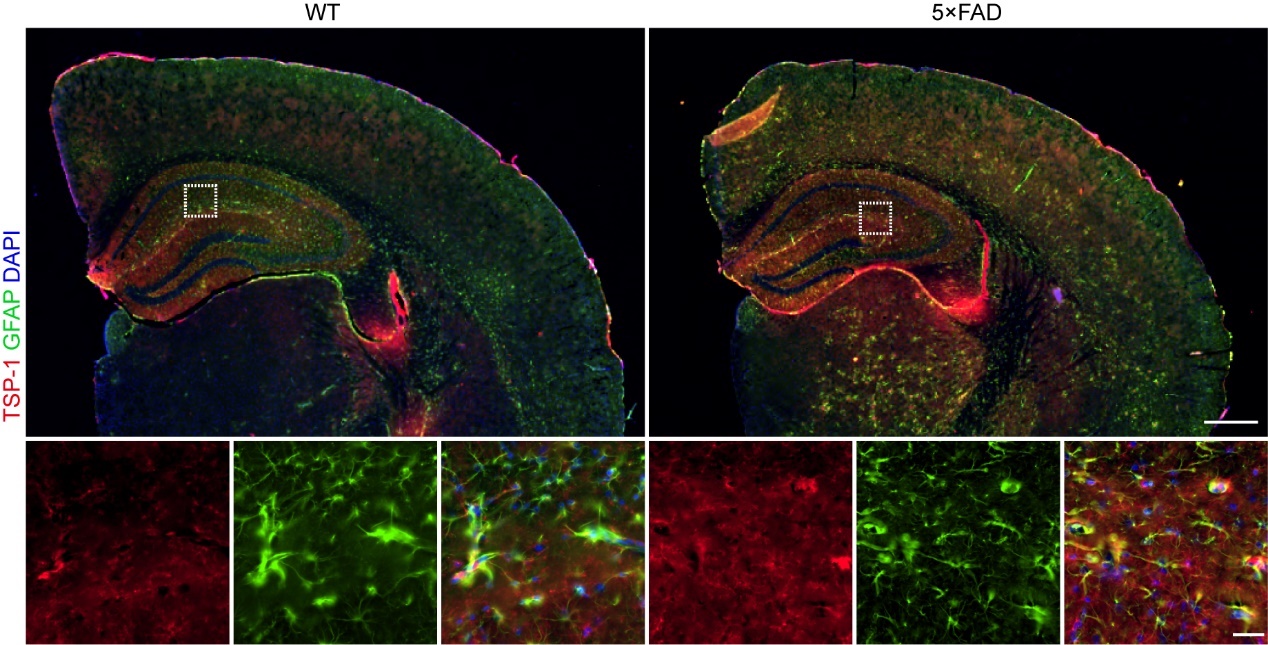


**Figure S3** TSP1 staining in mouse brain regions of WT and 5×FAD mice. Representative low magnification images of GFAP and TSP-1 staining. Scale bar, 500 μm (top) and 30 μm (bottom).


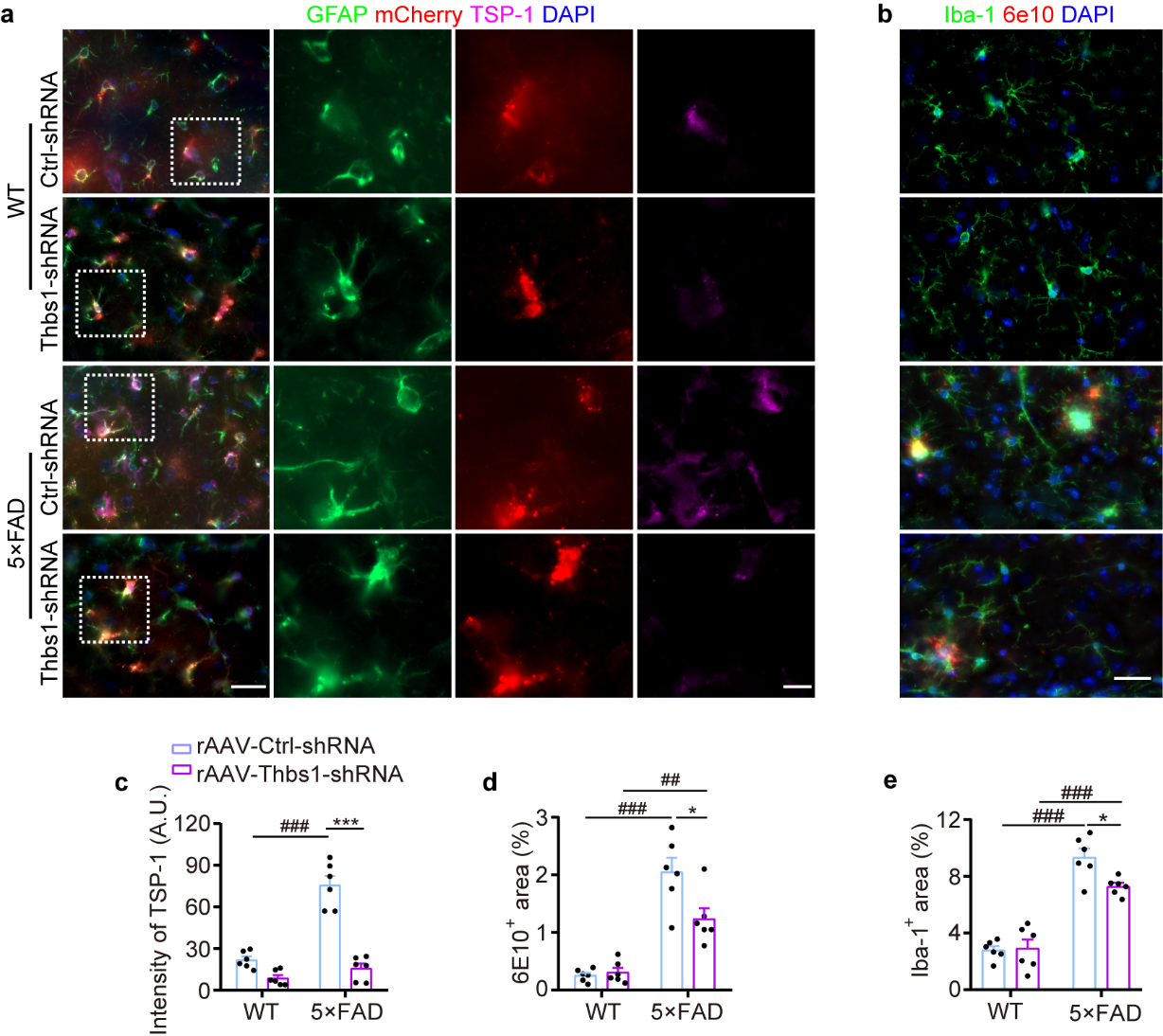


**Figure** **S4** Analysis of microglia and plaques after astrocyte-specific Thbs1 knockdown in the hippocampus of 5×FAD mice. **a, c** Representative images of GFAP, TSP-1 and mCherry staining (a) and quantification of the fluorescence intensity of TSP-1 (c) show the effect of astrocyte-specific TSP-1 knockdown in the hippocampus (n = 6 per group). Scale bar, 30 μm (left) and 10 μm (right). **b**, **d-e** Representative images of 6E10 and Iba-1 staining (b) and quantification of the percentage of 6E10^+^ (d) and Iba-1^+^ area (e) in the hippocampus, respectively (n = 6 per group). Scale bar, 30 μm. Data represent the mean ± SEM; significance was evaluated with two-way ANOVA with Tukey post hoc test. ***p < 0.001, AAV-ctrl-shRNA vs AAV-Thbs1-shRNA, ###p < 0.001, WT vs 5×FAD.


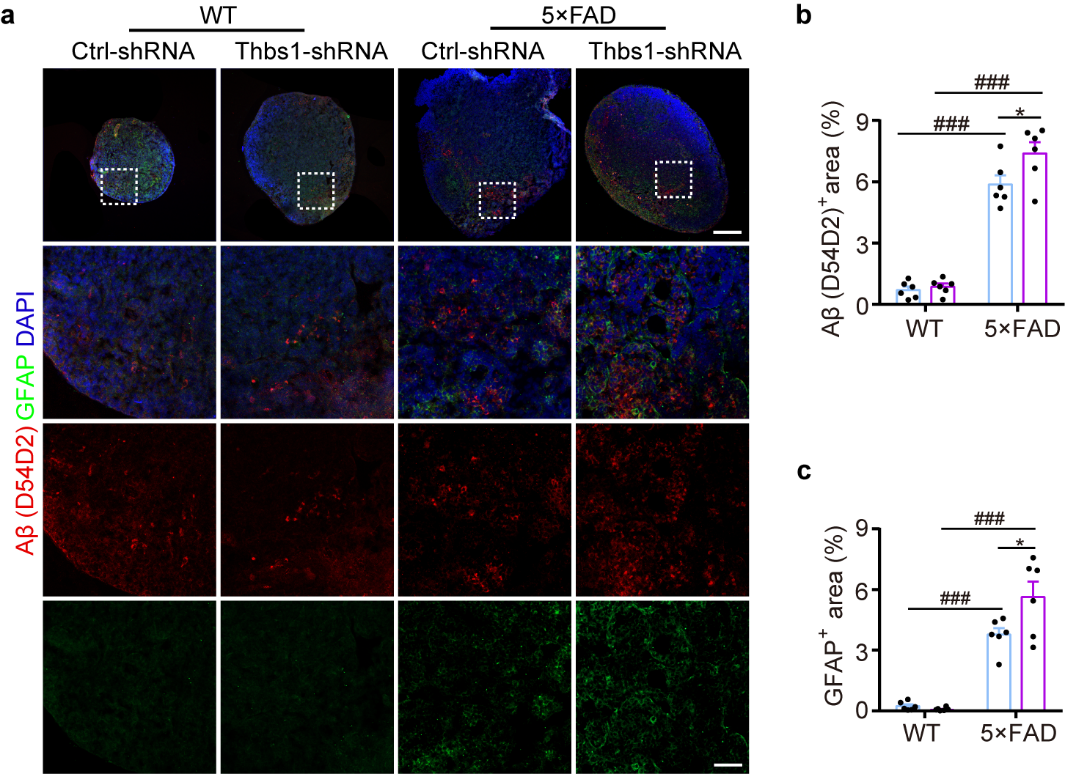


**Figure S5** Astrocyte-specific Thbs1 knockdown increased GFAP and Aβ levels in the dCLNs of 5×FAD mice. **a** Representative images of Aβ and GFAP staining in the dCLNs. Scale bar, 200 μm (left) and 40 μm (right). **b-c** Quantification of Aβ (b) and GFAP positive signals (c) in the dCLNs (n = 6 per group). Data represent the mean ± SEM; significance was evaluated with two-way ANOVA with Tukey post hoc test. *p < 0.05, AAV-ctrl-shRNA vs AAV-Thbs1-shRNA, ###p < 0.001, WT vs 5×FAD.


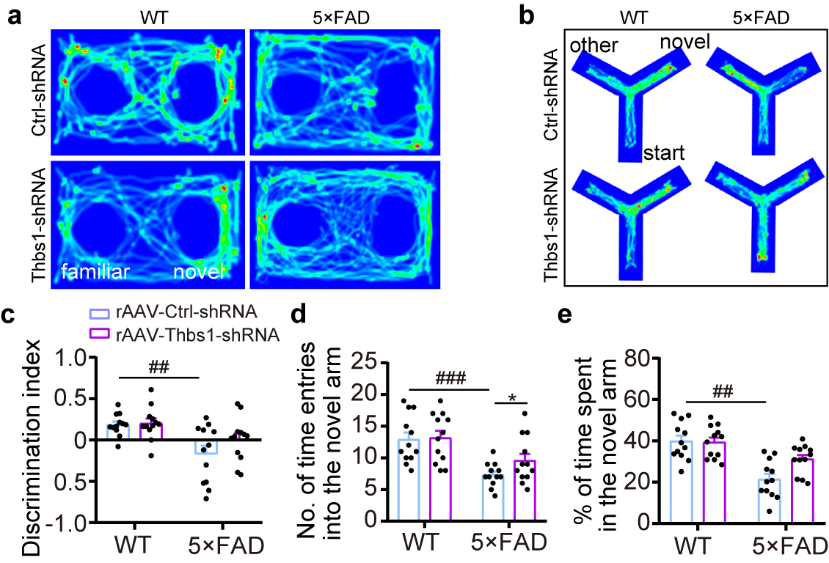


**Figure S6** Astrocyte-specific Thbs1 knockdown alleviated cognitive impairment of 5×FAD mice. **a, c** Movement tracing in the NOR test (a) and quantification of discrimination index (c) (n = 12 per group). **b, d-e** Movement tracing in the Y-maze test (b) and quantification of the percentage of the number of entries (d) and the percentage of time spent in the novel arm (e) (n = 12 per group). Data represent the mean ± SEM; significance was evaluated with two-way ANOVA with Tukey post hoc test. *p < 0.05, AAV-ctrl-shRNA vs AAV-Thbs1-shRNA, ##p < 0.01, ###p < 0.001, WT vs 5×FAD.


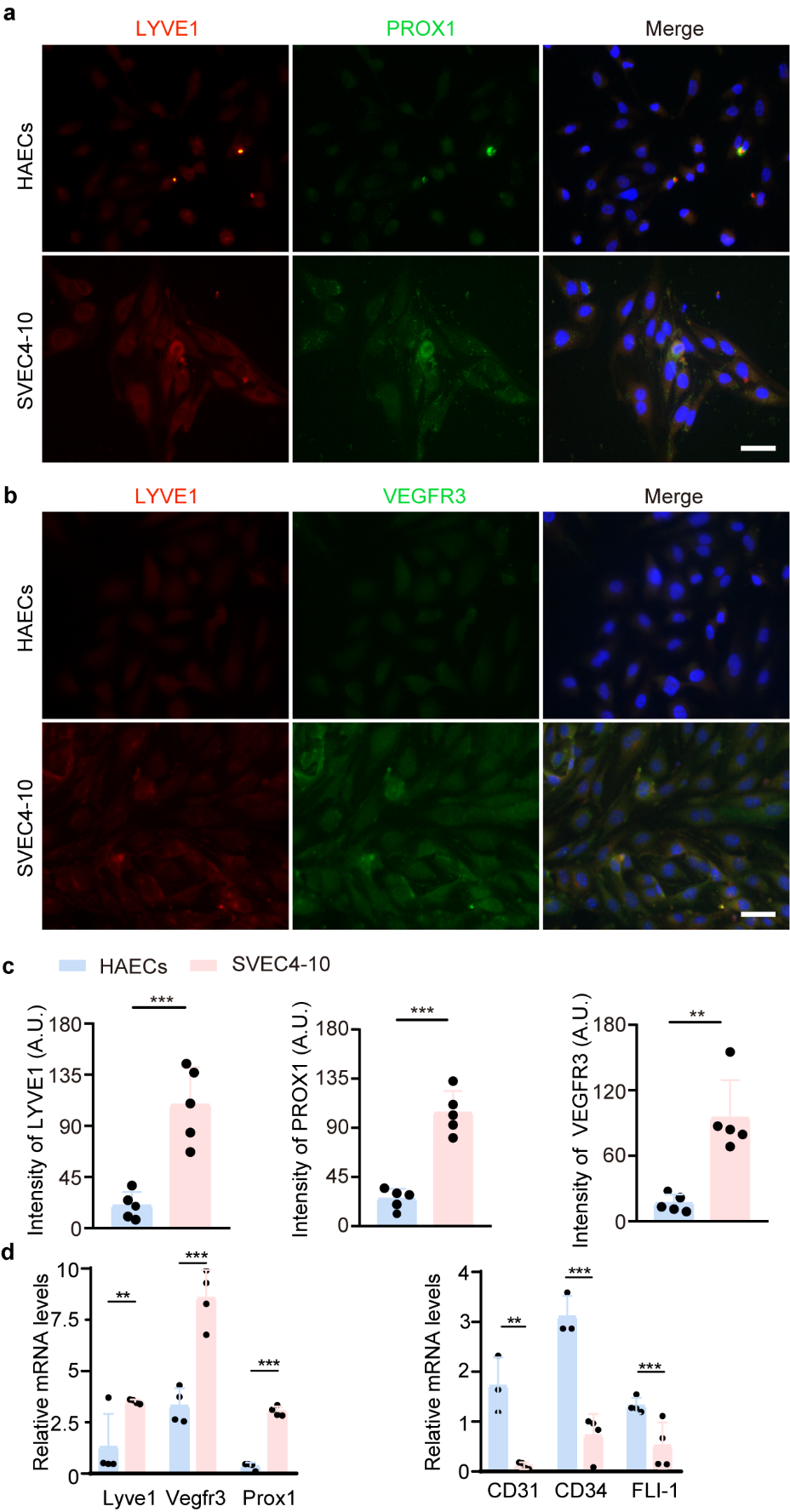


**Figure S7** Comparative identification of lymphatic endothelial cell lines of SVEC4-10 and vascular endothelial cell lines of HAECs. **a-c** Representative images of lymphatic endothelial markers labeled with LYVE1 and PROX1(a) and LYVE1 and VEGFR3 (b) on HAECs and SVEC4-10 cells and quantification of the fluorescence intensity of LYVE1, PROX1 and VEGFR3 (c) (n = 5 per group). Scale bar, 40 μm. **d** Relative mRNA levels of *Lyve1*, *Vegfr3*, *Prox1*, *CD31*, *CD34* and *FLI1* in both HAECs and SVEC4-10 cells (n = 3-4 per group). Data represent the mean ± SEM; significance was evaluated with one-way ANOVA with Tukey post hoc test or unpaired Student’s t-test (**p < 0.01, ***p < 0.001).


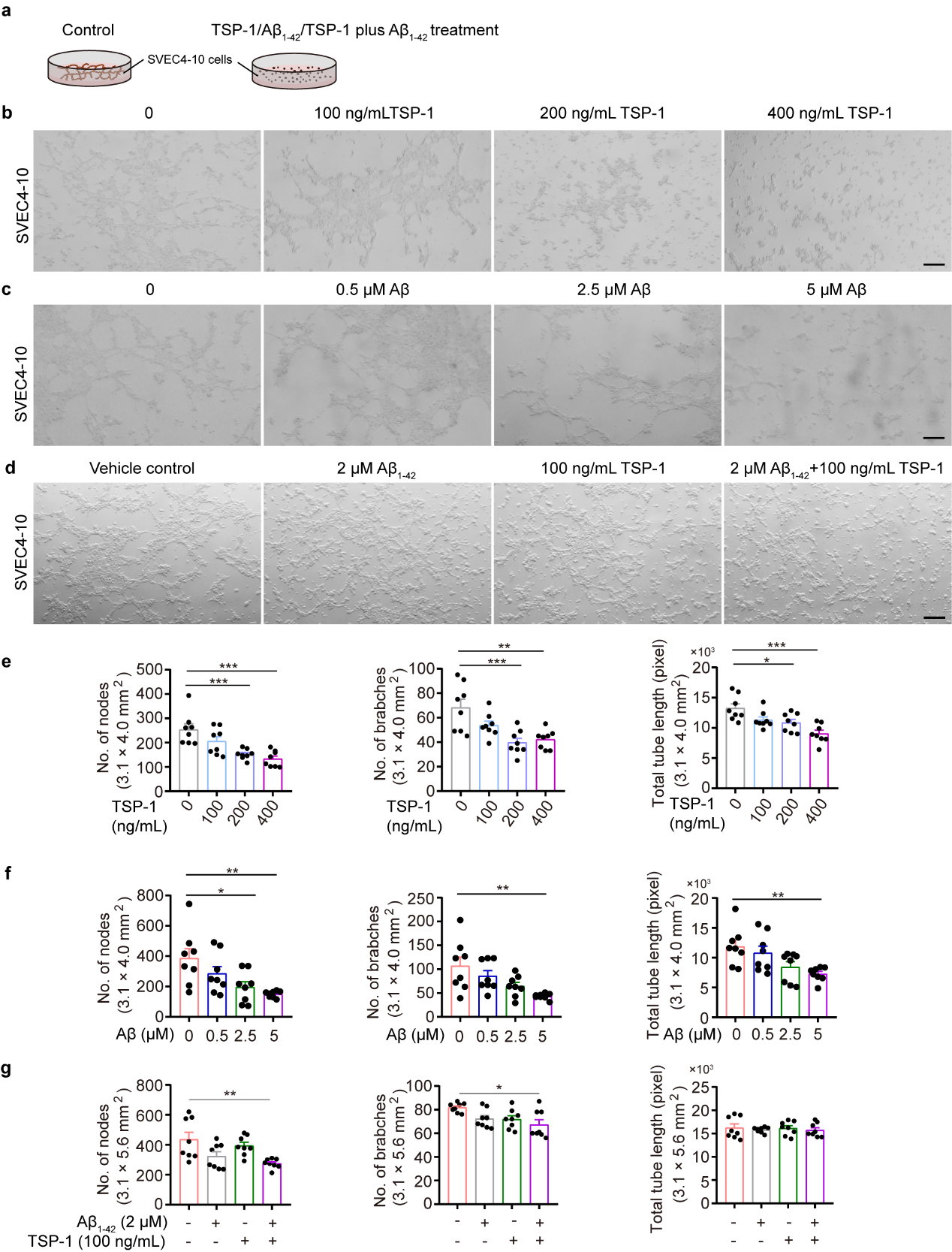


**Figure S8** The inhibitory of TSP-1 and Aβ on lymphatic vessel formation and plasticity via SVEC4-10 cells *in vitro*. **a** Schematics for tube formation in the SVEC4-10 cells pre-treated Aβ_1-42_ and exogenous recombinant TSP-1. **b, e** Representative images of tube formation assay pre-treated gradient concentrations of exogenous recombinant TSP-1 (b) and quantification of the numbers of nodes, sprouts and total tube length in random fields (3.1 × 4.0 mm^2^) (e). Scale bar, 100 μm, n = 8 per group **c, f** Representative images of tube formation assay in the SVEC4-10 cells pre-treated gradient concentrations of oligomeric Aβ (c) and quantification of the numbers of nodes, sprouts and total tube length in random fields (3.1 × 4.0 mm^2^) (f). Scale bar, 100 μm, n = 8 per group. **d, g** Representative images of tube formation assay in the SVEC4-10 cells pre-treated exogenous Aβ_1-42_ and recombinant TSP-1 (d) and quantification of the numbers of nodes, sprouts and total tube length in random fields (3.1 × 5.6 mm^2^) (g). Scale bar, 100 μm, n = 8 per group. Data represent the mean ± SEM; significance was evaluated with one-way ANOVA with Tukey post hoc test (*p < 0.05, **p < 0.01, ***p < 0.001).

**
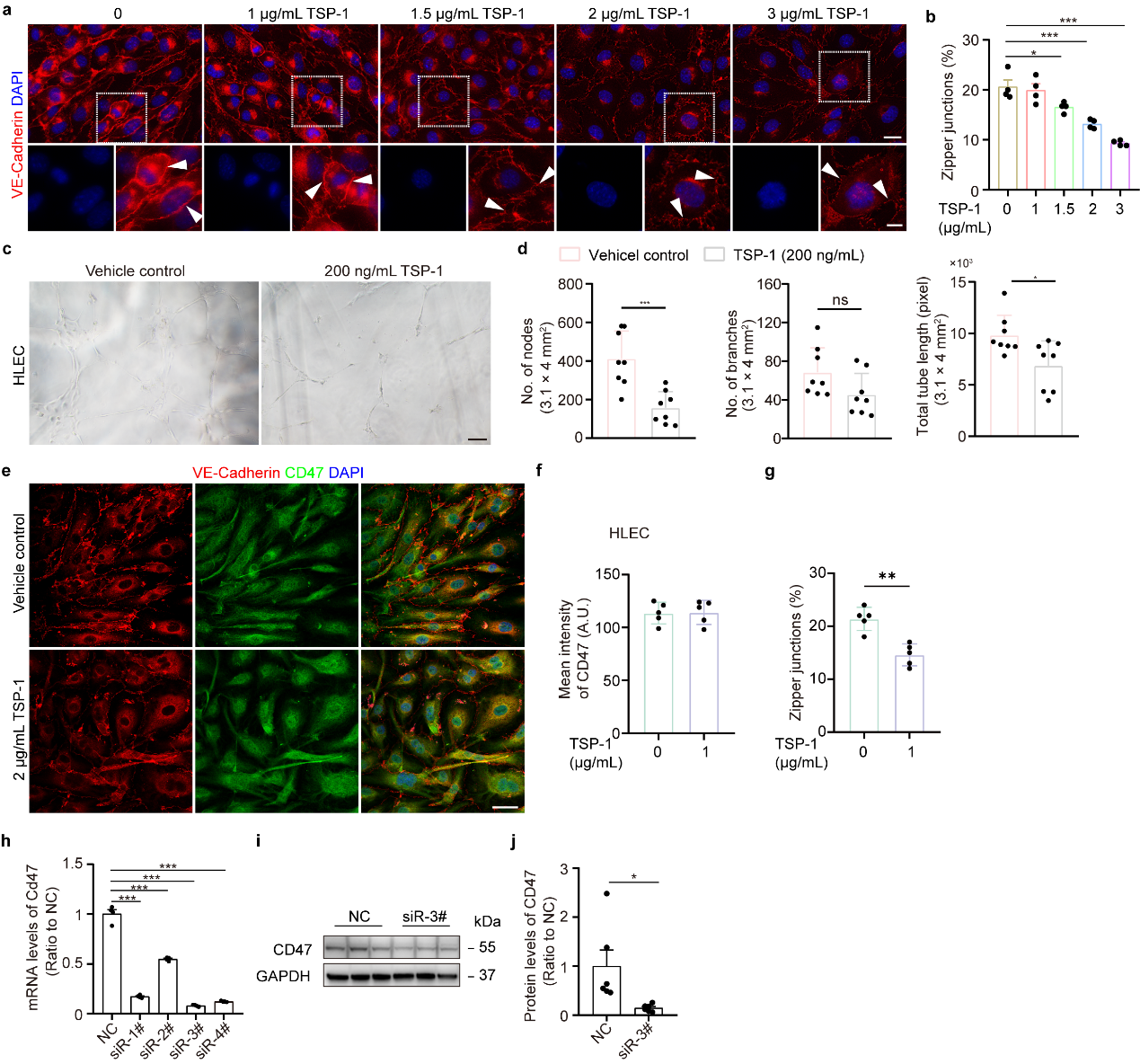
**

**Figure S9** TSP-1 dose-dependently inhibited VE-Cadherin-formed zipper-like junctions *in vitro*. **a-b** Representative images of VE-Cadherin staining in the SVEC4-10 cells pre-treated gradient concentrations of exogenous recombinant TSP-1 (a) and quantification of the percentage of zipper-like junctions (b). Scale bar, 20 μm (top) and 10 μm (bottom), n = 4 per group. **c, d** Representative images of tube formation assay pre-treated gradient concentrations of exogenous recombinant TSP-1 on HLEC (b) and quantification of the numbers of nodes, sprouts and total tube length in random fields (3.1 × 4.0 mm^2^) (e). Scale bar, 100 μm, n = 8 per group **e** Representative images of VE-Cadherin and CD47 staining in the HLEC cells. Scale bar, 50 μm. **f-g** Quantification of the fluorescence intensity of CD47 and percentage of zipper-like junctions (n = 5 per group). **h** Relative mRNA levels of Cd47 in SVEC4-10 cells with siRNA transfections for a knockdown in gene expression (n = 4 per group). **i-j** Representative Western blot bands (i) and densitometry analysis (j) of CD47 expression from SVEC4-10 cells with siRNA transfections for knockdown in gene expression (n = 6 per group). Data represent the mean ± SEM; significance was evaluated with one-way ANOVA with Tukey post hoc test (**b**, **h**, *p < 0.05, ***p < 0.001) or unpaired Student’s t-test (**d**, **f**, **g**, **j**, *p < 0.05).


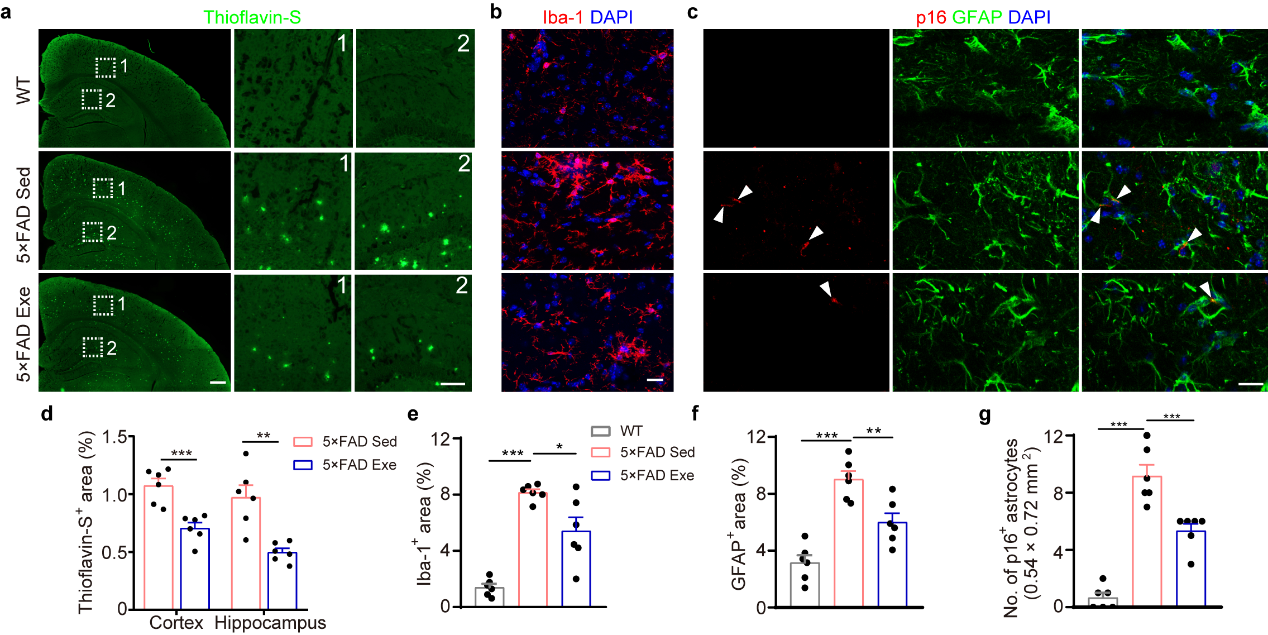


**Figure S10** Treadmill exercise alleviated deposition of Aβ, reactive microglosis and astrocyte senescence of 5×FAD mice. **a**, **d** Representative images of Thioflavin-S staining (a) and quantification of the positive area of Aβ plaques in the hippocampus and its surrounding cortical area (d). Scale bar, 400 μm (top) and 100 μm (bottom), n = 6 per group. **b, e** Representative images of ionized calcium-binding adaptor molecule 1 (Iba-1) staining (b) and quantification of the percentage of Iba-1 positive area in the hippocampus (e). Scale bar, 20 μm, n = 6 per group. **c, f-g** Representative images of senescent astrocytes (white arrowheads) characterized by highly expressing p16 in the hippocampal LMol (c) and quantification of the percentage of GFAP positive area (f) and the number of senescent astrocytes in the LMol (g). Scale bar, 20 μm, n = 6 per group. Data represent the mean ± SEM; significance was evaluated with one-way ANOVA with Tukey post hoc test. *p < 0.05, **p < 0.01, ***p < 0.001.


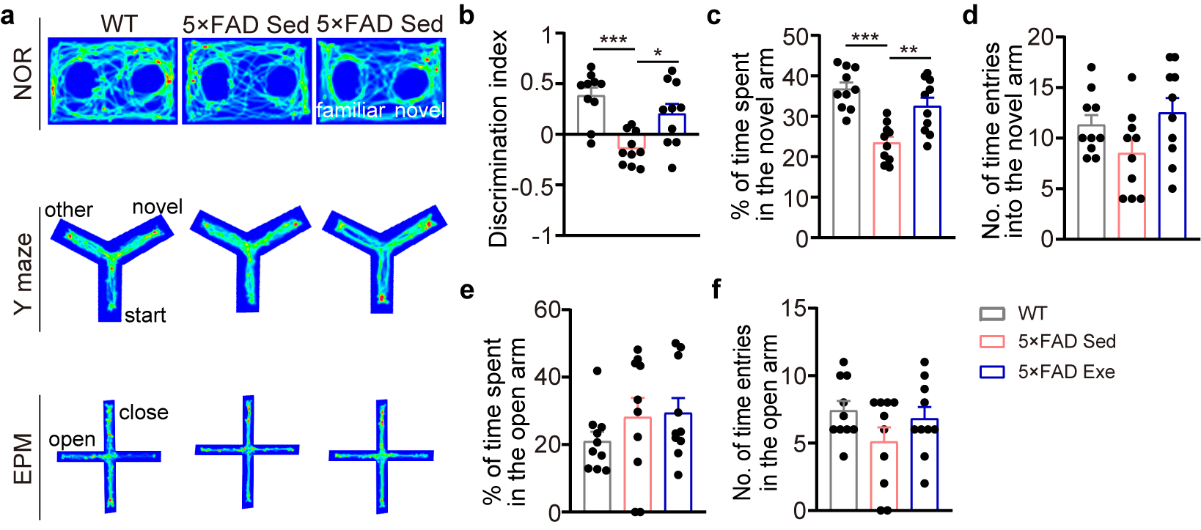


**Figure S11** Treadmill exercise alleviated cognitive deficits of 6.5-month-old 5×FAD mice. **a** Movement tracing in the NOR, Y maze and EPM test. **b** Quantification of discrimination index (n = 10 per group). **c**-**d** Quantification of the percentage of time spent in the novel arm (c) and the number of entries into the novel arm (d) (n = 10 per group). **e-f** Quantification of the percentage of time spent in the open arm (e) and the number of entries into the open arm (f) (n = 10 per group). Data represent the mean ± SEM; significance was evaluated with one-way ANOVA with Tukey post hoc test. *p < 0.05, **p < 0.01, ***p < 0.001.

**
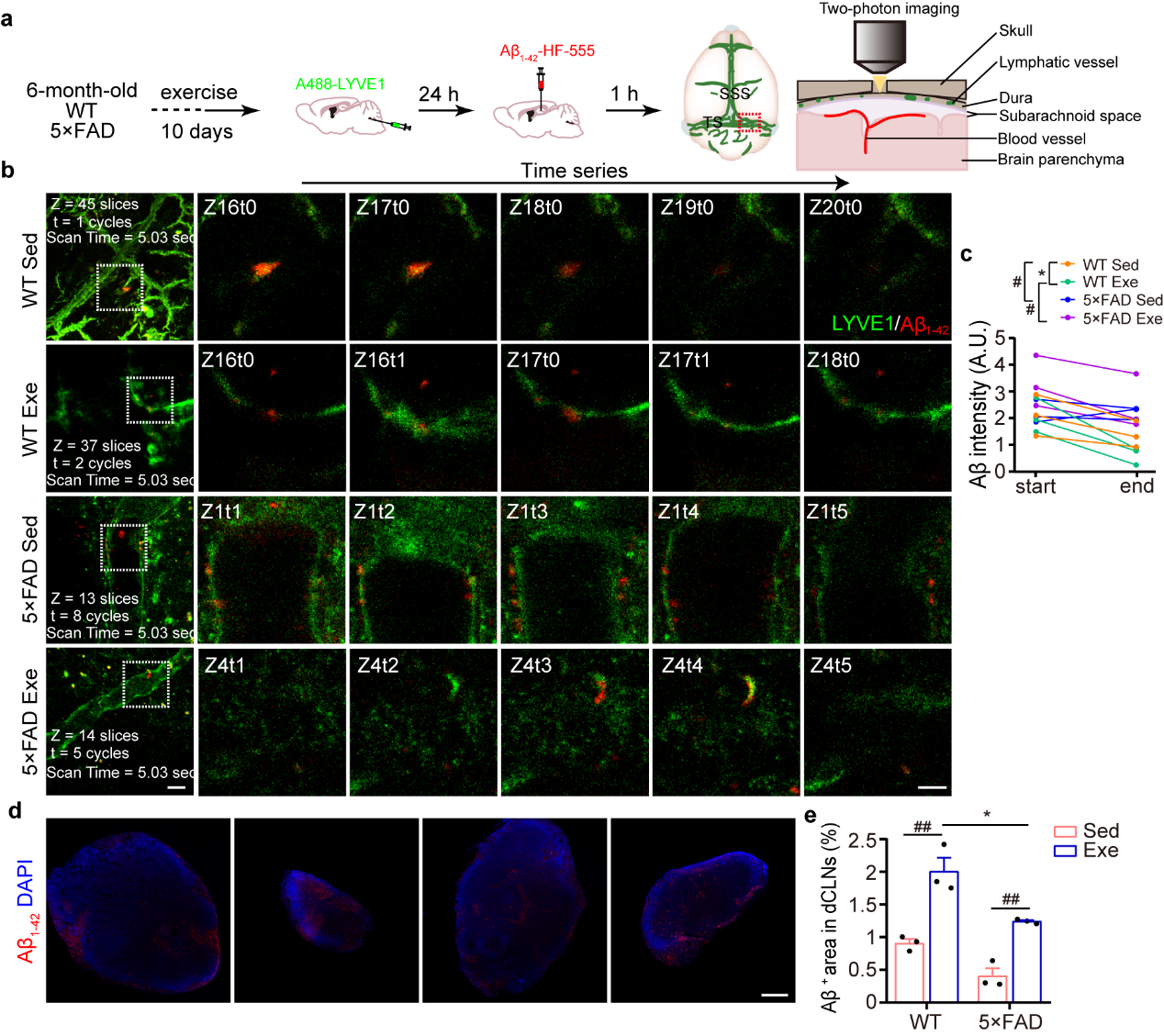
**

**Fig. S12** Treadmill exercise enhanced meningeal lymphatic vessels function to drain Aβ of 6.5-month-old 5×FAD mice. **a** Schematic of *in vivo* imaging in 6-month-old 5×FAD mice after administration of Aβ_1-42_-555 with treadmill training for 10 days. **b-c** Representative images (b) and quantification (c) of the fluorescence intensity in arbitrary units (A.U.) of Aβ_1-42_-555 in meningeal lymphatic vessels were captured at different time points after 1 hour of hippocampus injection with Aβ_1-42_-555 (n = 3 per group). Scale bar, 20 μm (left) and 10 μm (right). **d-e** Representative images (d) and quantification (e) of Aβ_1-42_-555 positive area in the dCLNs (n = 3 per group). Scale bar, 200 μm. Data represent the mean ± SEM; significance was evaluated with two-way ANOVA with Tukey post hoc test. *p < 0.05, Sed vs Exe, #p < 0.05, ##p < 0.01, WT vs 5×FAD.

**
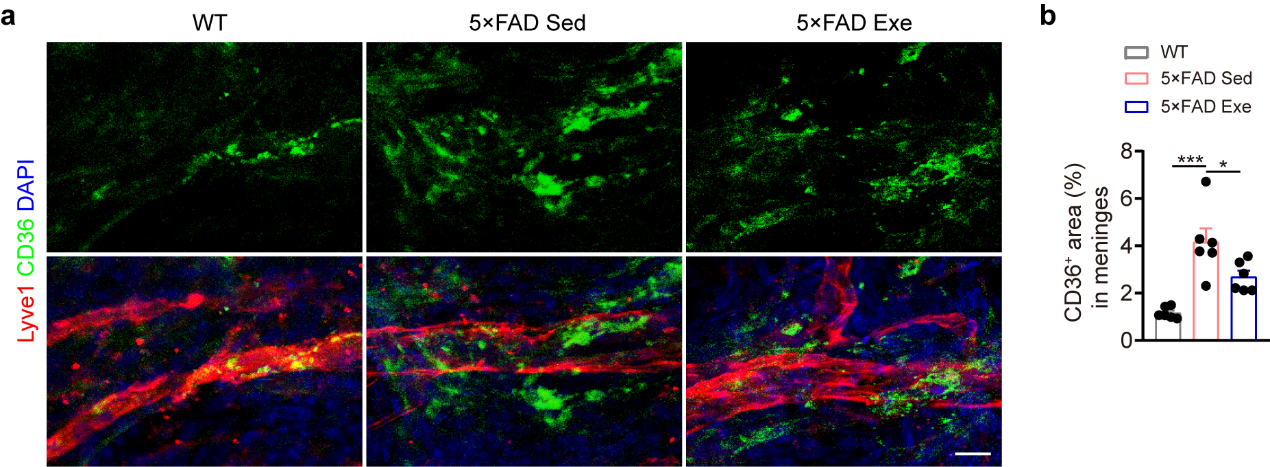
**

**Fig. S13** Treadmill exercise down-regulated the elevated CD36 levels in the meninges of 6.5-month-old 5×FAD mice. **a** Representative images of CD36 and Lyve1 staining (a) and quantification of the positive area of CD36 in the meninges (b). Scale bar, 50 μm, n = 6 per group. Data represent the mean ± SEM; significance was evaluated with one-way ANOVA with Tukey post hoc test. *p < 0.05, ***p < 0.001.


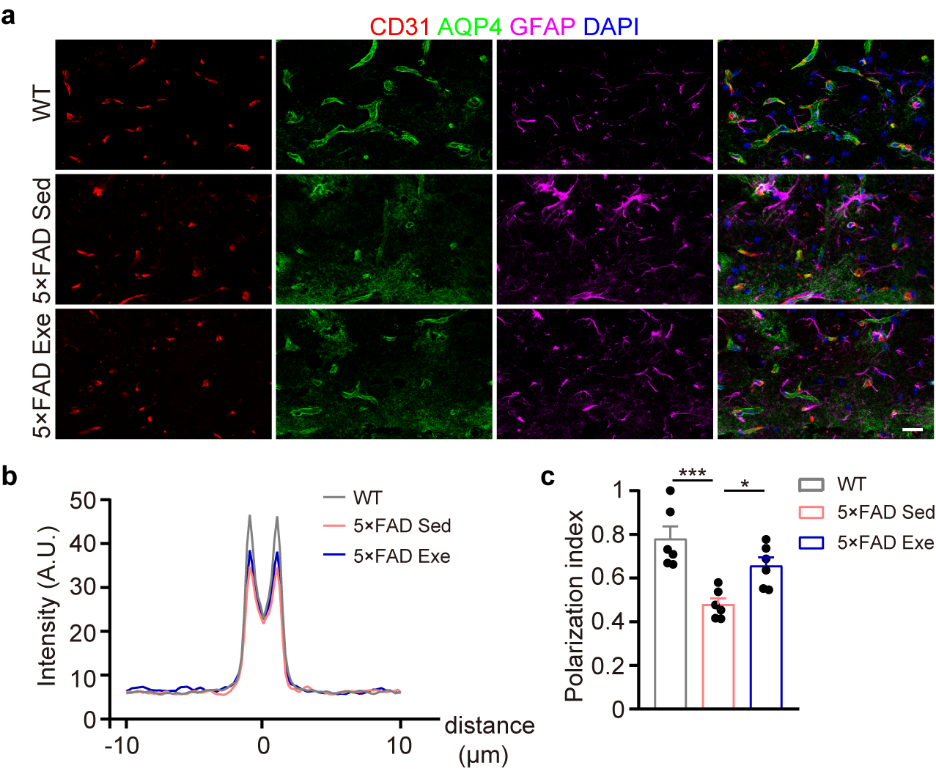


**Figure S14** Treadmill exercise improved perivascular AQP4 localization of 6.5-month-old 5×FAD mice. **a** Representative images of CD31, AQP4 and GFAP staining in hippocampal LMol area. Scale bar, 20 μm. **b** The average intensity of AQP4 staining in the hippocampus. **c** Boxplot displays the average polarization index of AQP4. Polarization index = Peak fluorescence of AQP4 positive perivascular endfeet – baseline. All values were normalized to the maximum signal for ease of visualization (n = 6 per group). Data represent the mean ± SEM; significance was evaluated with one-way ANOVA with Tukey post hoc test. *p < 0.05, ***p < 0.001.


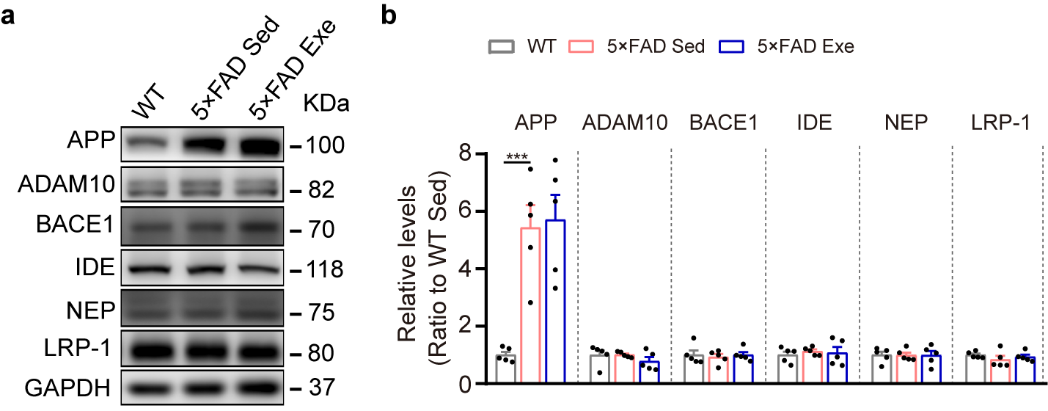


**Figure S15** Analysis of treadmill exercise on Aβ production and clearance-related enzyme of 6.5-month-old 5×FAD mice. **a-b** Representative Western blot bands (a) and densitometry analysis (b) of APP, ADAM10, BACE1, IDE, NEP, and LRP-1 levels in the hippocampus of 6.5-month-old WT and 5×FAD mice (n = 5 per group). Data represent the mean ± SEM; significance was evaluated with one-way ANOVA with Tukey post hoc test. ***p < 0.001.


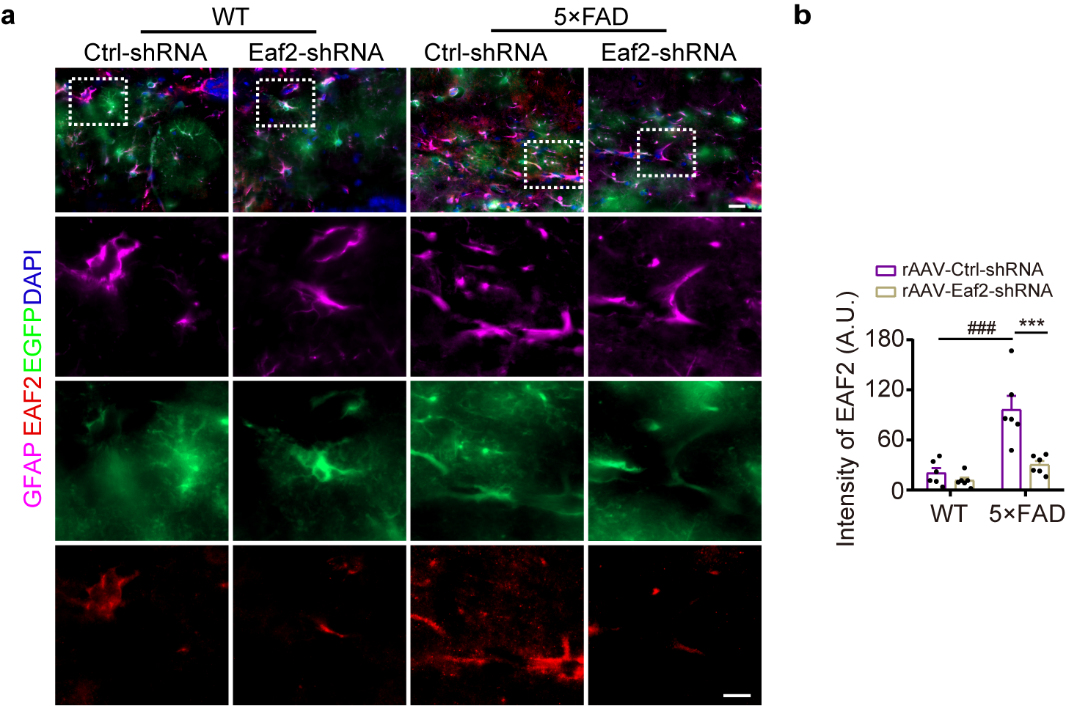


**Figure S16** Astrocyte-specific Eaf2 knockdown in the hippocampus of 5×FAD mice. **a** Representative images of GFAP, EAF2 and EGFP staining in the hippocampus. Scale bar, 20 μm (left) and 10 μm (right). **b** Quantification of the fluorescence intensity of EAF2 showing the effect of astrocyte-specific Eaf2 knockdown (n = 6 per group). Data represent the mean ± SEM; significance was evaluated with two-way ANOVA with Tukey post hoc test. ***p < 0.001, AAV-ctrl-shRNA vs AAV-Eaf2-shRNA, ###p < 0.001, WT vs 5×FAD.


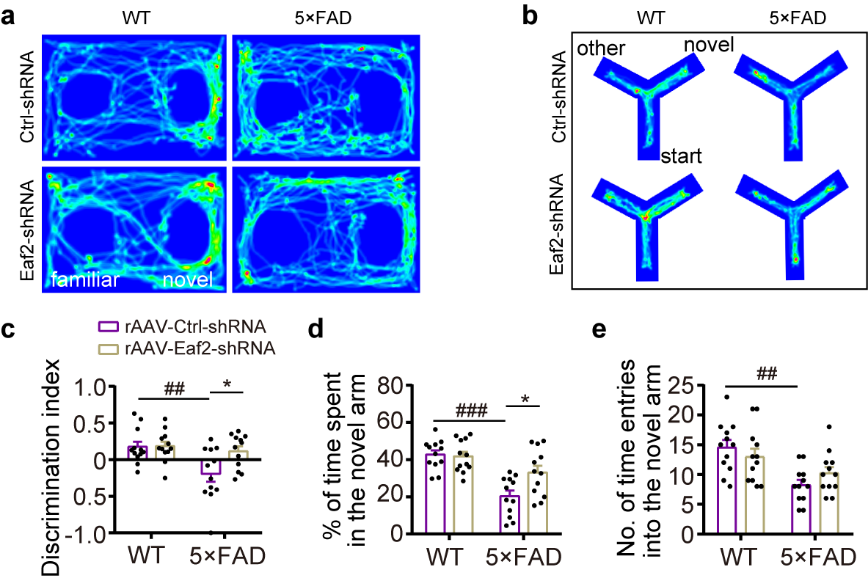


**Figure S17** Astrocyte-specific Eaf2 knockdown alleviated cognitive impairment of 5×FAD mice. **a**, **c** Movement tracing in the NOR test (a) and quantification of discrimination index (c) (n = 12 per group). **b**, **d-e** Movement tracing in the Y-maze test (b) and quantification of the percentage of the number of entries (d) and the percentage of time spent in the novel arm (e) (n = 12 per group). Data represent the mean ± SEM; significance was evaluated with two-way ANOVA with Tukey post hoc test. *p < 0.05, AAV-ctrl-shRNA vs AAV-Eaf2-shRNA, ##p < 0.01, ###p < 0.001, WT vs 5×FAD.


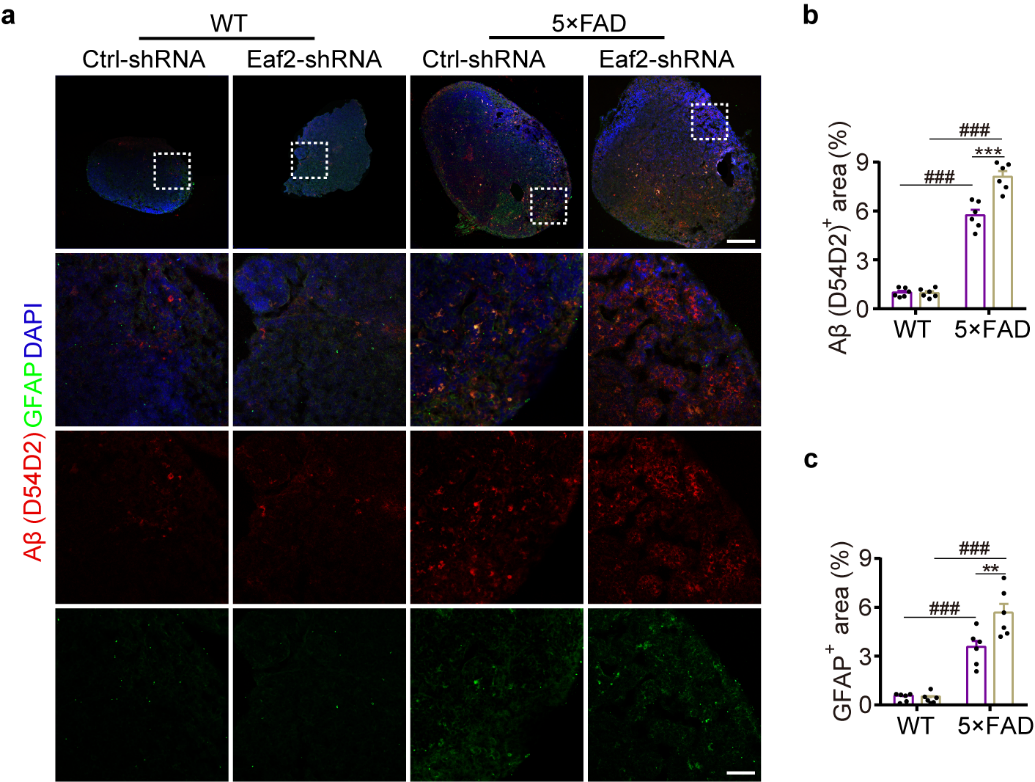


**Figure S18** Astrocyte-specific Eaf2 knockdown increased GFAP and Aβ levels in the dCLNs of 5×FAD mice. **a** Representative images of Aβ, GFAP staining in the dCLNs. Scale bar, 200 μm (top) and 40 μm (bottom). **b-c** Quantification of Aβ positive area (b) and GFAP positive signals (c) in the dCLNs (n = 6 per group). Data represent the mean ± SEM; significance was evaluated with two-way ANOVA with Tukey post hoc test. **p < 0.01, ***p < 0.001, AAV-ctrl-shRNA vs AAV-Eaf2-shRNA, ###p < 0.001, WT vs 5×FAD.

**Table S1: antibodies used in this study**

| Antibodies | Source | Catalog number | RRID | Species | Dilution  IHC/IF | WB |
| --- | --- | --- | --- | --- | --- | --- |
| 6E10 | Biolegend | 803001 | AB_2564653 | mouse | 1:600 |  |
| APP | Sigma | A8717 | AB_258409 | rabbit |  | 1:1000 |
| AQP4 | Millipore | AB3594 | AB_91530 | rabbit | 1:400 |  |
| BACE1 | Millipore | MAB5308 | AB_95207 | rabbit |  | 1:1000 |
| CD31 | RD Systems | AF3628 | AB_2161028 | goat | 1:200 |  |
| CD36 | Santa cruz | Sc-7309 | AB_627044 | mouse | 1:200 |  |
| CD47 | Proteintech | 66304-1-1g | N/A | mouse | 1:400 | 1:3000 |
| CD68 | Abcam | Ab53444 | AB_869007 | rat | 1:200 |  |
| EAF2 | Biorbyt | Orb412548 | N/A | rabbit |  | 1:1000 |
| EAF2 | Proteintech | 11172-1-AP | AB_2097560 | rabbit | 1:100 | 1:1000 |
| GAPDH | Proteintech | 60004-1-Ig | AB_2107436 | mouse |  | 1:3000 |
| GFAP | Millipore | MAB360 | AB_11212597 | mouse | 1:800 | 1:1000 |
| GFAP | Abcam | Ab4674 | AB_304558 | chicken | 1:800 |  |
| GFAP | Abcam | Ab279291 | N/A | rat | 1:800 |  |
| Iba-1 | Wako | 019-19741 | AB_839504 | rabbit | 1:600 |  |
| IDE | Abcam | Ab32216 | AB_775686 | rabbit |  | 1:1000 |
| LRP-1 | Abcam | Ab92544 | AB_2234877 | rabbit |  | 1:1000 |
| LYVE-1 | Abcam | Ab33682 | AB_881387 | rabbit | 1:250 |  |
| NEP | Millipore | AB5458 | AB_2144423 | rabbit |  | 1:1000 |
| p16 | Abcam | Ab54210 | AB_881819 | mouse | 1:250 |  |
| P53 | Santa cruz | Sc-126 | AB_628082 | mouse | 1:400 | 1:1000 |
| PROX1 | Abcam | Ab199359 | AB_2868427 | rabbit | 1:200 |  |
| S100β | Abcam | Ab52642 | AB_882426 | rabbit | 1:600 |  |
| Trem2 | Abcam | Ab245227 | N/A | rabbit | 1:200 |  |
| TSP-1 | Proteintech | 18304-1-AP | AB_2201959 | rabbit | 1:200 | 1:1000 |
| TSP-1 | Santa cruz | Sc-59887 | AB_793045 | mouse | 1:200 |  |
| VE-Cadherin | Abcam | Ab205336 | AB_2891001 | rabbit | 1:500 |  |
| Human VE-Cadherin | RD Systems | AF938 | N/A | goat | 1:100 |  |
| β-tublin | Proteintech | 10094-1-AP | AB_1072210 | rabbit |  | 1:8000 |
| β-Amyloid (D54D2) | Cell Signaling Technology | 8243 | AB_2830152 | rabbit | 1:600 |  |
| AF488-conjugated anti-mouse IgG | ThermoFisher | A21202 | AB_141607 | donkey | 1:1000 |  |
| AF488-conjugated anti-rabbit IgG | ThermoFisher | A21206 | AB_2535792 | donkey | 1:1000 |  |
| AF488-conjugated anti-chicken IgG | Abcam | ab150169 | AB_141607 | goat | 1:1000 |  |
| AF488-conjugated anti-rat IgG | Abcam | ab150153 | AB_2737355 | donkey | 1:1000 |  |
| AF555-conjugated anti-rabbit IgG | ThermoFisher | A31572 | AB_162543 | donkey | 1:1000 |  |
| AF555-conjugated anti-mouse IgG | ThermoFisher | A31570 | AB_2536180 | donkey | 1:1000 |  |
| AF555-conjugated anti-goat IgG | ThermoFisher | A21432 | AB_2535853 | donkey | 1:1000 |  |
| AF647-conjugated anti-rabbit IgG | ThermoFisher | A32795TR | AB_2866496 | donkey | 1:1000 |  |
| AF647-conjugated anti-mouse IgG | ThermoFisher | A31571 | AB_162542 | donkey | 1:1000 |  |

**Table S2**: qRT-PCR primer information

| Primer | Primer sequence (5' to 3') |
| --- | --- |
| Mus Atf1-F | GTGAGGAGTCTCAGGACTC |
| Mus Atf1-R | CTGCTCGTCTGATAGATGG |
| Mus Cd31-F | CTCCCAGAACATGGATGTGGAGTA |
| Mus Cd31-R | GTGCATCTGCCTTGGCTGTC |
| Mus Cd34-F | GGTAAGCTCTCTGCCTGATGAG |
| Mus Cd34-R | TGGTAGGAACTGATGGGGATATT |
| Mus Cd47-F | TGGTGGGAAACTACACTTGCG |
| Mus Cd47-R | CGTGCGGTTTTTCAGCTCTAT |
| Mus Egr1-F | CAGTGGCAACACTTTGTGG |
| Mus Egr1-R | AGTAGATGGGACTGCTGTC |
| Mus Fli-1-F | ATACGGATTGATGGAGATTG |
| Mus Fli-1-R | GGTAACTGAGGTGCGACA |
| Mus Fosl1-F | ACCTTGTGCCAAGCATCGA |
| Mus Fosl1-R | TCTCACCCTGCGGCGCTCTT |
| Mus Gapdh-F | GGTTGTCTCCTGCGACTTCA |
| Mus Gapdh-R | TGGTCCAGGGTTTCTTACTCC |
| Mus Id1-F | TAGCTGTTCGCTGAAGGC |
| Mus Id1-R | TCGTAGAGCAGGACGTTCA |
| Mus Lyve1-F | GGAATTCGCACAATGGTCCAGCACACTAGCCT |
| Mus Lyve1-R | GCTCTAGATTCTCCACTCTCTTTGCATCTACCAT |
| Mus P53-F | CTCTCCCCCGCAAAAGAAAAA |
| Mus P53-R | CGGAACATCTCGAAGCGTTTA |
| Mus Prox1-F | TGGCTTATCCATTTCAGAGTC |
| Mus Prox1-R | CTTCACGTCCGAGAAGTAGG |
| Mus Runx2-F | AGTCCCAACTTCCTGTGC |
| Mus Runx2-R | CGGCGGAGTAGTTCTCAT |
| Mus Vegfr3-F | TGGACAGCTGGACGGAGTTT |
| Mus Vegfr3-R | CTGGCAGAGGAGTTTACGCA |
| Homo CD31-F | CCTGCGGTATTCAAAGACAA |
| Homo CD31-R | TGGGACCAGATCCTTCATTCAC |
| Homo CD34-F | CAAACATCACAGAAACGACAGT |
| Homo CD34-R | GGACAGAAGAGTTTGTGTTTCC |
| Homo FLI-1-F | GGATGGCAAGGAACTGTGTAA |
| Homo FLI-1-R | GGTTGTATAGGCCAGCAG |
| Homo GAPDH-F | ATGGGGAAGGTGAAGGTCG |
| Homo GAPDH-R | GGGTCATTGATGGCAACAATA |
| Homo LYVE1-F | CTGCTGGAGGAAAAGTCTGG |
| Homo LYVE1-R | GTCTTGATGTCTGCGTGGG |
| Homo PROX1-F | GCCTAATGGTCTTTCATTCTGC |
| Homo PROX1-R | TGGCCTTACAGGTCGAATTC |
| Homo VEGFR3-F | GTGGGTGGGTAGTGAGGAGA |
| Homo VEGFR3-R | AGGTCATCTTCGTCGTCCC |
