# Supplementary figures and images for: Long-term exercise enhances meningeal lymphatic vessel plasticity and drainage in a mouse model of Alzheimer's disease
Source: Transl Neurodegener. 2025 Jul 25;14:37. doi: 10.1186/s40035-025-00497-2 (PMC12291319; doi:10.1186/s40035-025-00497-2)

**Fig. 6d**


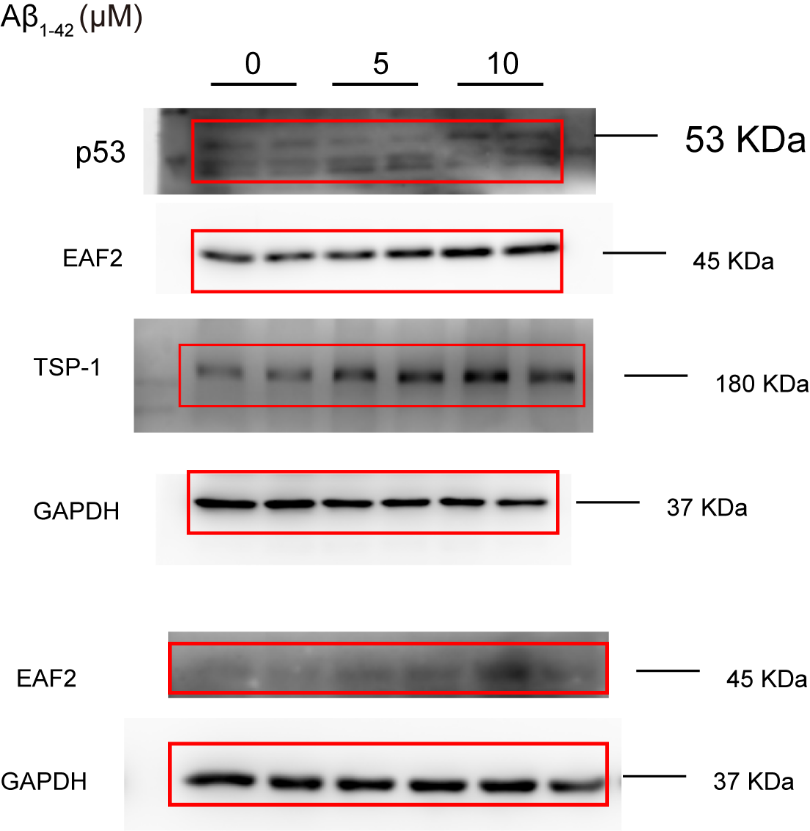


**Fig. 7d**


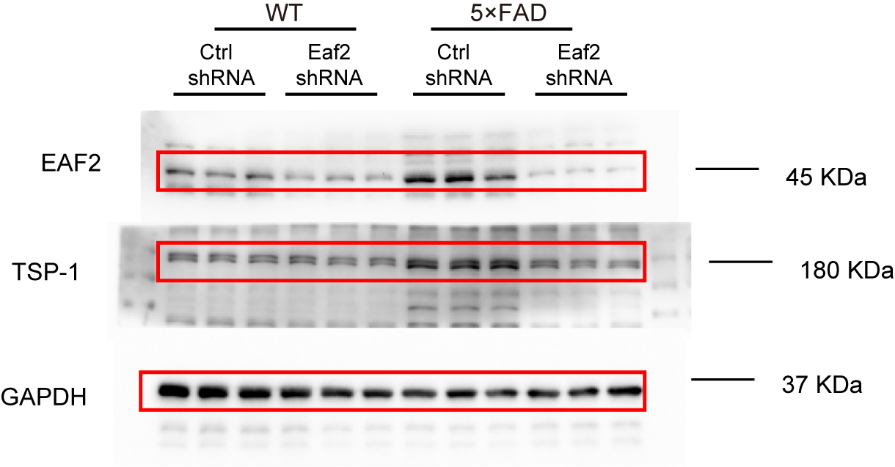


**Fig. S9i**


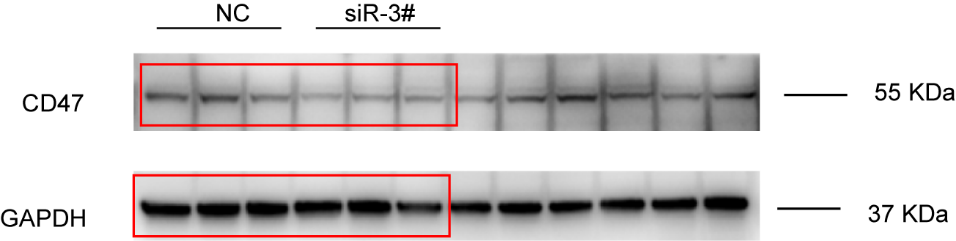


**Fig. S15a**


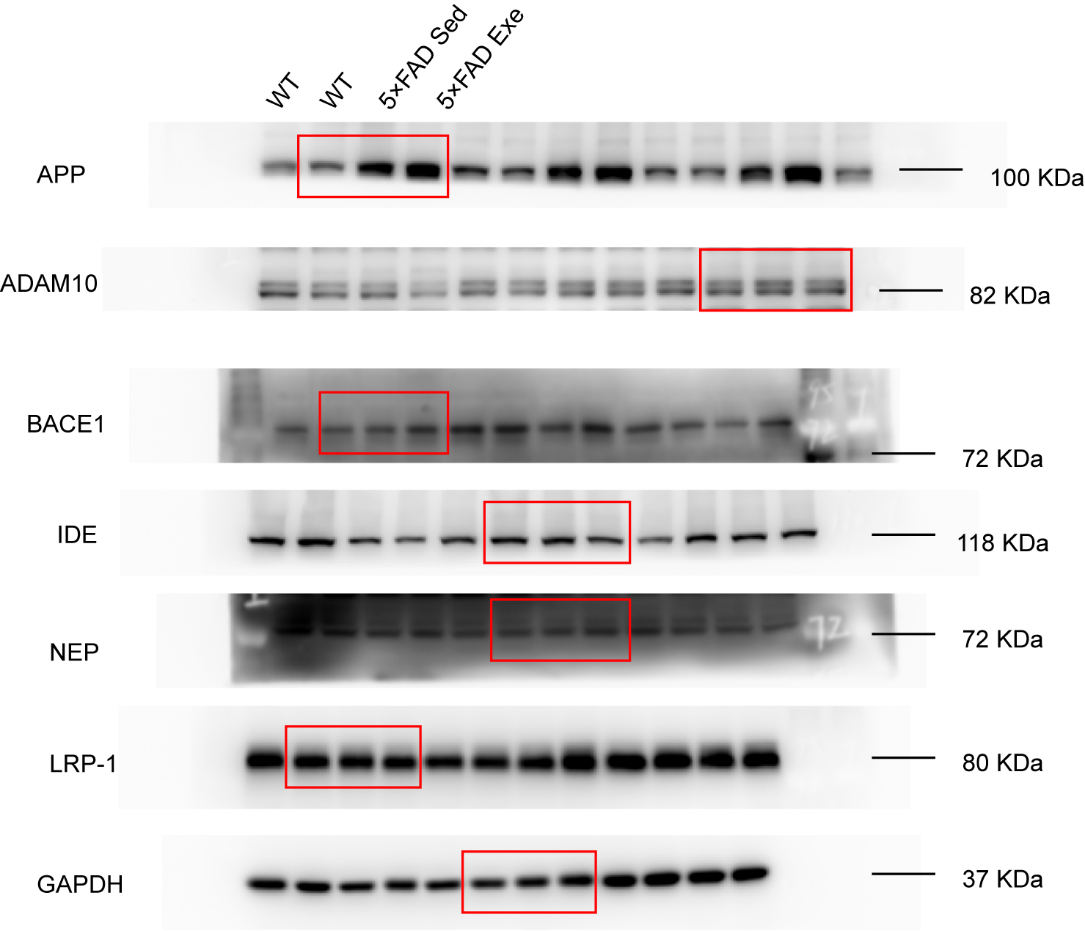

Supplement: Supplementary file 2 — Additional file 2: Original western blot bands [file 40035_2025_497_MOESM2_ESM.docx]
